# Achieving strong optical nonlinearity and wide bandgap of pnictides via ionic motif–driven directed assembly of covalent groups

**DOI:** 10.1126/sciadv.adr2389

**Published:** 2024-11-13

**Authors:** Lihua Gao, Jindong Chen, Xuemei Shi, Yan Xiao, Yinglei Han, Chensheng Lin, Huikang Jiang, Guangsai Yang, Guang Peng, Ning Ye

**Affiliations:** ^1^ Tianjin Key Laboratory of Functional Crystal Materials, Institute of Functional Crystal, College of Materials Science and Engineering, Tianjin University of Technology, Tianjin 300384, China.; ^2^ Tianjin Key Laboratory of Quantum Optics and Intelligent Photonics, School of Science, Tianjin University of Technology, Tianjin 300384, China.; ^3^ College of Chemistry and Materials Science, Hebei University, Baoding, Hebei 071002, China.; ^4^ MOE Key Laboratory of Weak-Light Nonlinear Photonics, School of Physical Sciences, Nankai University, Tianjin 300457, China.; ^5^ Key Laboratory of Optoelectronic Materials Chemistry and Physics, Fujian Institute of Research on the Structure of Matter, Chinese Academy of Sciences, Fuzhou, Fujian 350002, China.

## Abstract

Noncentrosymmetric (NCS) pnictides are indispensable for nonlinear optics, ferroelectrics, magnetic Weyl electronics, etc., areas, yet their structure design remains a substantial challenge. By using asymmetric ionic unit–driven covalent groups orienting and rigidity-flexibility coupling dual strategy, we successfully design and synthesize four NCS pnictides: [Sr_4_Br]_2_[M^II^_3_Si_25_P_40_] (M^II^ = Mg, Cd) and [Ba_3_Br][M^III^Si_10_P_16_] (M^III^ = Ga, In), which exhibit strong second harmonic generation effects (5.2 to 7.5 × AgGaS_2_), wide bandgaps (1.81 to 1.90 electron volts), and moderate birefringence (0.030 to 0.051). An unprecedented NCS structure-inducing mechanism analysis revealed that the (Sr_4_Br) and (Ba_4_Br) ionic units featuring the diamond-like electrostatic force field effectively break inversion symmetry and trigger uniform arrangement of the covalent tetrahedron groups. Furthermore, the nonlinear optical (NLO) properties and birefringence can be remarkably tuned by the secondary covalent building blocks (M^II/III^P_4_ tetrahedra) with distinct bond flexibility providing a broader space for regulating the key parameters. This work might expand chemical space for exploiting high-performance pnictide NLO materials.

## Teaser

Diamond-like electrostatic field of ionic motifs trigger the codirectional arrangement of covalent tetrahedron groups.

## INTRODUCTION

Inorganic pnictides with superconductive, nonlinear optical (NLO), thermoelectric, transport, catalytic, etc., properties have attracted widespread interest and sustained attention (*1*–*6*). Pnictide with noncentrosymmetric (NCS) and polar structure are crucial material mediums for nonlinear optics, ferroelectrics, magnetic Weyl electronics, etc., research fields (*7*–*9*). Now, the main pnictide-based NLO materials, ZnGeP_2_, CdSiP_2_, CdGeAs_2_ and OP-GaP/GaAs, exhibit superior NLO properties than their chalcogenide analogs, e.g., AgGaS_2_, AgGaSe_2_, ZnS, CdSe, etc. (*10*, *11*). However, because of some intrinsic drawbacks, their application scenarios are severely limited. Great breakthroughs in areas of science rely heavily on the great discovery of new high-performance materials. High-performance infrared (IR) NLO crystals require balanced regulation of frequency conversion efficiency, laser damage threshold, bandgap, and birefringence. Pnictides generally have large NLO coefficients and wide IR transmission ranges, which are excellent IR NLO crystal candidates (*12*).

**Fig. 1.**
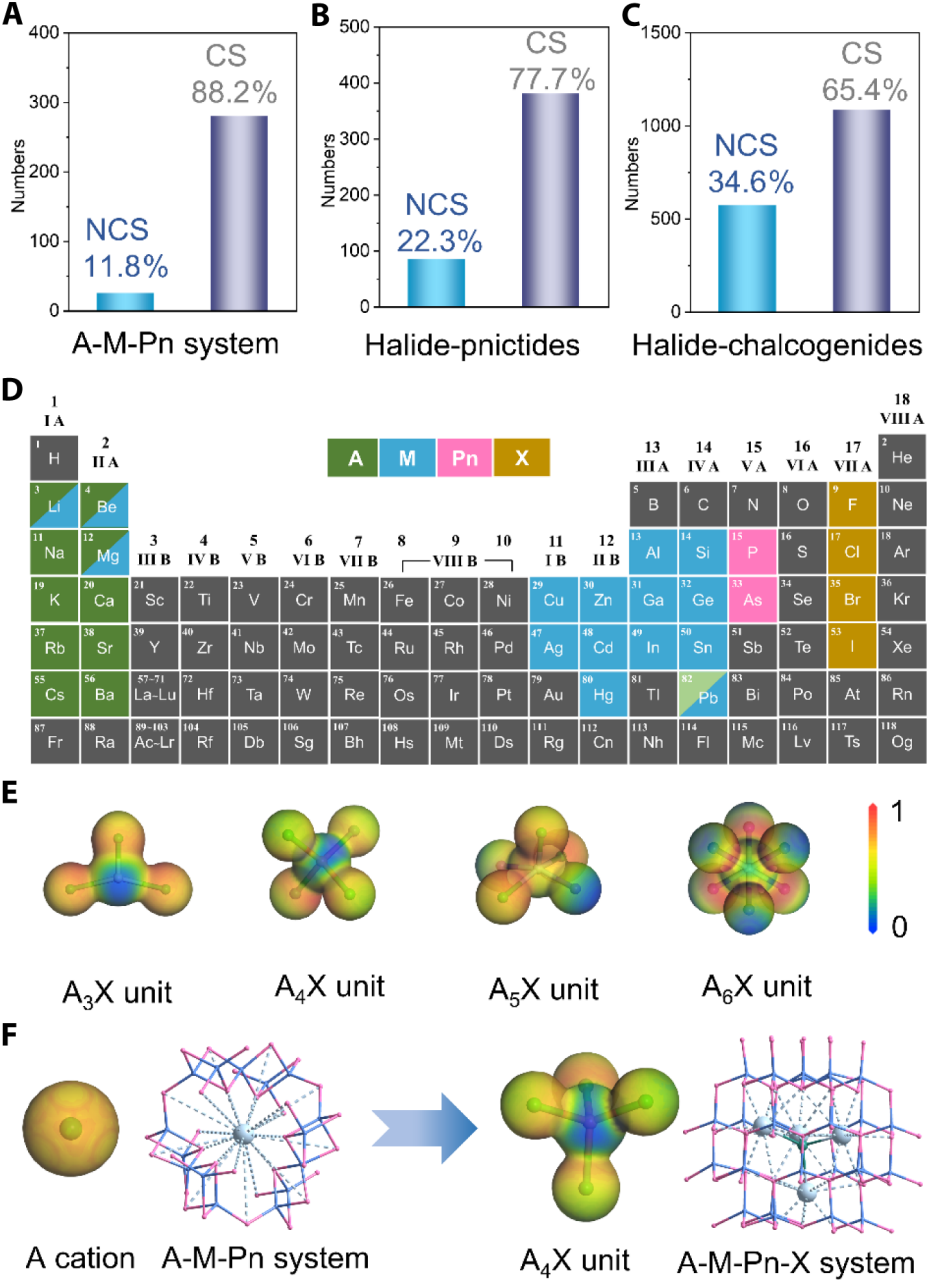
NCS and CS structure statistics and electrostatic potential scheme. (**A** to **C**) Proportion of pnictides with NCS and CS structure in the A-M-Pn system (A: K, Rb, Cs, Sr, and Ba; M: IIB, IIIA, and IVA elements; Pn: P and As), halide-pnictides (pnictogens: P and As; halogens: Cl, Br, and I), halide-chalcogenides (chalcogens: S and Se; halogens: Cl, Br, and I), respectively. (**D**) Chemical space of A-M-Pn-X phase fields. (**E**) Electrostatic potential of asymmetric A_3_X, A_4_X, A_5_X, and symmetric A_6_X units. (**F**) Tetrahedron arrangement scheme under electrostatic interactions of spherical A cation and asymmetric A_4_X.

However, pnictides suffer from scarce NCS structures and narrow optical bandgap. No inversion symmetry is the prerequisite for a second-order NLO effect. The bandgap not only is directly proportional to the laser damage threshold but also determines the shortest wavelength of the pump laser source that can be used. Combining the strongly electropositive alkali and alkaline earth metals with the NLO-active group to balance the bandgap and optical nonlinearity is the conventional design idea for new inorganic NLO crystals (*13*–*18*). Unexpectedly, this NLO material design paradigm cannot work well on obtaining high-performance NLO pnictides. Our previous work has revealed that the bandgap regulation of pnictides obeys a complex mechanism requiring systemic reconciliation of the ionicity, covalency, and metallicity (*19*). With the increase in radius and positivity of A-site alkali and alkaline earth metals, their contributions on bandgap change from positive to negative due to the decrease in ionic polarization and covalent nature between A-Pn interactions. Therefore, the vast majority of K-, Rb-, Cs-, Sr-, and Ba-pnictides in an A-M-Pn (A: group 1 and 2 elements; M: group 12, 13, and 14 elements; Pn: P and As) system have no wider bandgaps (mostly <2.0 eV) than Li, Mg, Na, and Ca analogs (table S8), although excluding no bandgap-detrimental group 11 elements (Cu, Ag, and Au) and early transition metals with unfilled d/f electron orbitals. In addition, because the spherical electrostatic interaction of A cations to neighboring Pn atoms always tend to induce symmetric arrangements of M-Pn groups, NCS structures are difficult to be achieved. According to our statistics based on the latest The International Centre for Diffraction Data (ICDD) database (PDF-5+ 2024), the proportion of pnictides crystallizing in NCS space groups among total 305 pnictides in the A-M-Pn system is only 8.2% (Fig. 1A). Does it mean we should give up exploring pnictide NLO crystals containing strongly ionic K, Rb, Cs, Sr, and Ba metals?

For efficiently designing NCS pnictides, we propose introducing halogens X into the A-M-Pn system (i.e., targeting A-M-Pn-X phase fields) to construct asymmetric A-X ionic units to break the local inversion symmetry of M-Pn groups. This expands that the potential material design space (Fig. 1D) for structure and performance control will be an effective approach but is rarely experimentally achieved. The proportion of pnictides with NCS structure in a halide-pnictide system has increased to 22.3%, which is 10% more than the A-M-Pn system (Fig. 1B). However, the number of halide-pnictides (the total number is 381) is far less than that of halide-chalcogenides (the total number is 1656), possibly indicating that it is seriously underexplored (Fig. 1C). In the A-M-Pn system, halogens usually coordinate with A cationic lattice to form A-X ionic units such as A_3_X, A_4_X, A_5_X, etc. (Fig. 1E), which can modulate covalent building blocks dominating the NLO properties. Attributed to the asymmetric electrostatic force field effect of asymmetric A-X ionic units, the inversion symmetry of the local structure is broken and the arrangement of covalent groups is directionally assembled (Fig. 1F). This has been partially verified in related oxides and chalcogenides, e.g., KSrCO_3_F, K_3_B_6_O_10_Cl, CsBa_3_B_2_S_6_I, [RbBa_2_Cl][Ga_4_S_8_], etc. (*20*–*23*). In addition, halogens can improve the ionic nature of structure and increase the bandgap. Furthermore, the “rigidity-flexibility coupling” strategy is adopted to construct covalent building blocks (*24*). The synergistic polarization effect of rigid (M1Pn_4_) and flexible (M2Pn_4_) tetrahedron groups are favorable to high NLO performances. Simultaneously, the large atomic radius difference between M1 and M2 can create certain lattice deformation and group distortion, contributing to moderate birefringence. Herein, the rigid SiP_4_ tetrahedron was selected elaborately as the M1Pn_4_ group due to its coordinated performance between the highest occupied molecular orbital–lowest unoccupied molecular orbital gap and hyperpolarizability favorable to the balance of optical bandgap and NLO coefficient (*19*).

Driven by considerations above, we successfully designed and synthesized four A-M-Pn-X system pnictides: [Sr_4_Br]_2_[Mg_3_Si_25_P_40_] (SBMSP), [Sr_4_Br]_2_[Cd_3_Si_25_P_40_] (SBCSP), [Ba_3_Br][GaSi_10_P_16_] (BBGSP), and [Ba_3_Br][InSi_10_P_16_] (BBISP). To date, the [KBa_6_Cl][Si_12_P_20_] family recently reported by Kovnir and colleagues (*25*) is the only known A-M-Pn-X phase field pnictides. Regrettably, they crystallize in the centrosymmetric (CS) space group of *Fm*$\bar{3}$*m* because highly symmetric Ba_6_Cl octahedron ionic motifs resulted in the inverse configuration of (Si_6_P_16_) covalent building blocks. Herein, all tetrahedron groups in title pnictides are orientationally assembled due to the diamond-like electrostatic induction of acentric (A_4_Br) ionic units. They achieved strong second harmonic generation (SHG) effects (5.2 to 7.5 × AgGaS_2_), wide bandgaps (1.81 to 1.90 eV), and moderate birefringence (0.030 to 0.051). The internal inducing mechanism of (A_4_Br) ionic units to covalent building blocks was elucidated from the perspectives of lattice topology and local coordination.

## RESULTS

### Crystal structure description and comparison

Title crystals were synthesized with the raw materials of SrBr_2_, BaBr_2_ (3N, Aladdin), Mg, Cd, Ga, In, Si, Li (3N, Adamas), and P (5N, Adamas) through a self-flux method (the experimental details are provided in Materials and Methods). The colors of obtained title crystals range from red to dark red (fig. S1). The good fitting of powder x-ray diffraction (PXRD) Rietveld refinement patterns suggested that the pure phase of the title compounds was obtained (fig. S2). Energy-dispersive spectroscopy analysis confirmed the presence of composition elements (fig. S3).

**Fig. 2.**
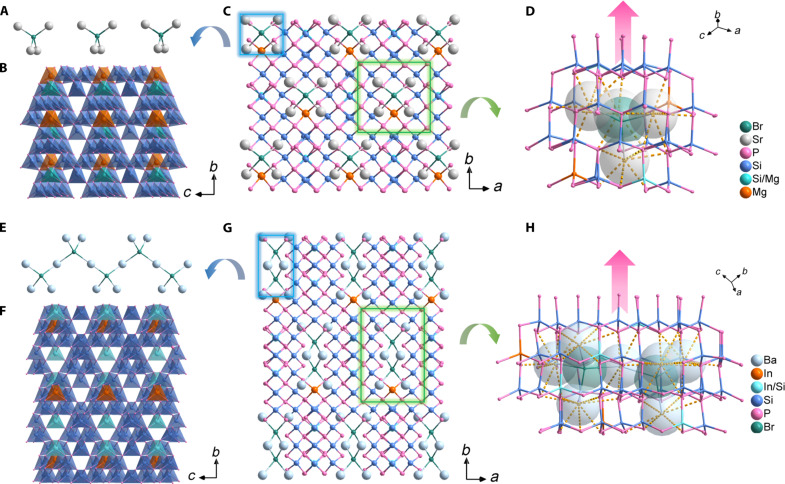
Crystal structures of SBMSP and BBISP. (**A**) ^0D^[Sr_4_Br]^7+^ ionic motifs. (**B**) [InSi_10_P_16_]^5−^ covalent building blocks (where blue, cyan, and brown represent SiP_4_, Mg/SiP_4_, and MgP_4_ tetrahedra, respectively). (**C**) Crystal structure of SBMSP viewed down the *c* axis. (**D**) Tetrahedra arrangement around [Sr_4_Br]^7+^ ionic motifs in SBMSP. (**E**) [Ba3Br]5+∞1D ionic motifs. (**F**) [Mg_3_Si_25_P_40_]^7−^ covalent building blocks (where blue, cyan, and brown represent SiP_4_, In/SiP_4_, and InP_4_ tetrahedra, respectively). (**G**) Crystal structure of BBISP viewed down the *c* axis. (**H**) Tetrahedra arrangement around [Ba3Br]5+∞1D ionic motifs in BBISP.

SBMSP and BBGSP are isomorphic with SBCSP and BBISP, respectively, which all crystallize in a polar space group *C*2. The crystal structures of SBMSP and BBISP were discussed as representatives. SBMSP and BBISP exhibit similar structure skeletons with a neat arrangement of covalent building blocks and ionic motifs (Fig. 2, C and G). In one asymmetric unit, there are 22 crystallography-independent sites including 2 Sr, 1 Br, 1 Mg, 1 Mg/Si, 7 Si, and 10 P atoms for SBMSP and 34 crystallography-independent sites including 3 Ba, 1 Br, 1 In, 2 In/Si, 11 Si, and 16 P atoms for BBISP. SBMSP and BBISP both exhibit a three-dimensional (3D) covalent open framework of [Mg_3_Si_25_P_40_]^7−^ and [InSi_10_P_16_]^5−^ with (Sr_4_Br) and (Ba_4_Br) units occupying the tunnels, respectively. The (Sr_4_Br) in SBMSP is isolated, i.e., ^0D^[Sr_4_Br]^7+^ motifs (Fig. 2A), while (Ba_4_Br) in BBISP is assembled into 1D infinite [Ba3Br]5+∞1D chain-like motifs (Fig. 2E) by sharing two Ba atoms. All tetrahedron groups including SiP_4_, Mg/SiP_4_, and MgP_4_ tetrahedra in SBMSP and SiP_4_, In/SiP_4_, and InP_4_ tetrahedra in BBISP are well directionally arranged (Fig. 2, B and F) like those in the diamond-like structure, attributed to the asymmetric electrostatic force field effect of diamond-like (A_4_Br) ionic units (Fig. 2, D and H). The detailed mechanism was discussed in the next paragraph. Moreover, all tetrahedron groups exhibit a significant polarization along the *b* axis (table S5), and the total intrinsic dipole moment within one unit cell is 14.4, 12.9, 22.7, and 7.6 D.

**Fig. 3.**
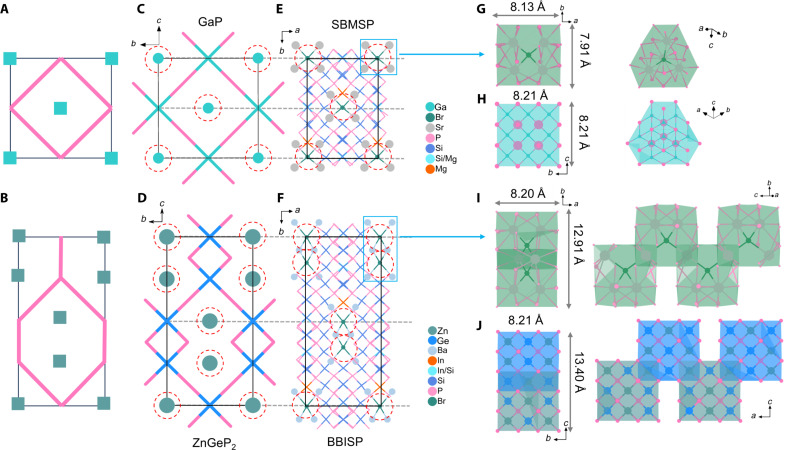
Structure comparison to GaP and ZnGeP_2_. (**A** and **B**) Modular patterns of SBMSP and BBISP. (**C** to **F**) Crystal structures of GaP, SBMSP, ZnGeP_2_, and BBISP. (**G**) (Sr_4_Br)@P_24_ polyhedrons in SBMSP. (**H**) (P_4_Ga_13_)@P_24_ cubic dodecahedron in GaP. (**I**) [Ba_6_Br_2_]@P_36_ jagged chains in BBISP. (**J**) [Zn_11_Ge_13_P_8_]@P_36_ jagged chains in ZnGeP_2_.

The structures of SBMSP and BBISP are significantly relevant to sphalerite GaP and chalcopyrite ZnGeP_2_, respectively, which both belong to diamond structure derivants. The (A_4_Br) ionic units in SBMSP and BBISP have consistent topology sublattice with marked Ga atoms in GaP and marked Zn atoms in ZnGeP_2_ (Fig. 3, C to F), and the spatial distribution of covalent groups has a very high topological similarity to (GaP_4_) groups in GaP and (GeP_4_) groups in ZnGeP_2_, respectively.

**Fig. 4.**
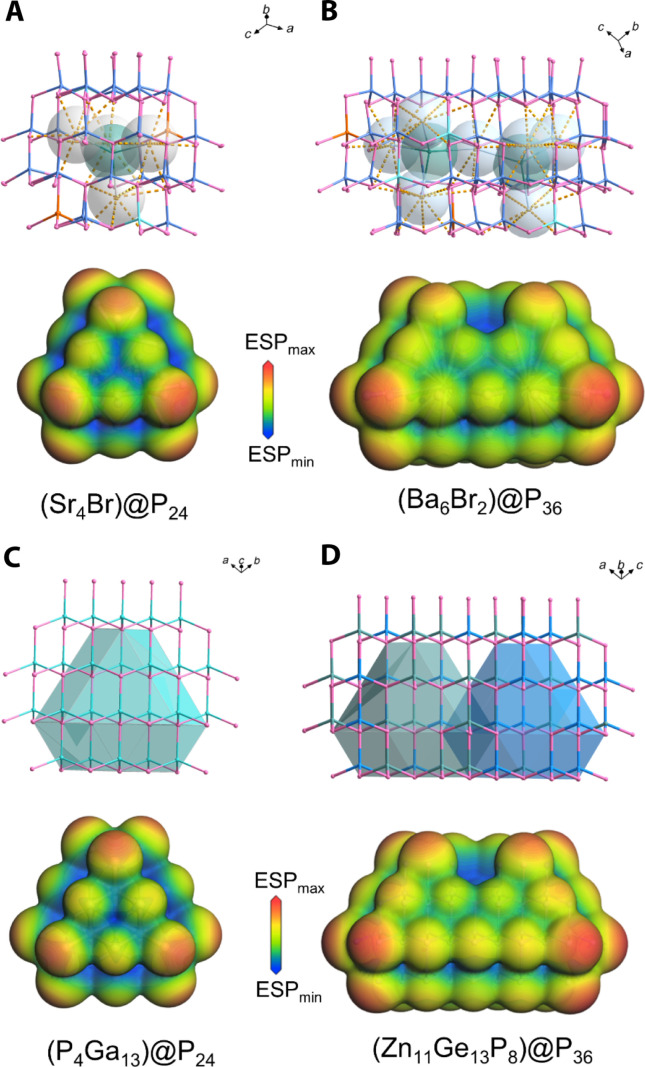
Tetrahedron group arrangement and electrostatic potential diagrams. (**A** to **D**) Orderly arrangement of circumambient tetrahedron groups and calculated electrostatic potential (ESP) diagram mapping on the electron density of (Sr_4_Br)@P_24_, [Ba_6_Br_2_]@P_36_, (P_4_Ga_13_)@P_24_, and [Zn_11_Ge_13_P_8_]@P_36_.

As depicted in modular patterns (Fig. 3, A and B), the structures of SBMSP and GaP, BBISP, and ZnGeP_2_ can be described by the same modular feature. Because all tetrahedra in the diamond-like structure are parallelly arranged, it can be reasonably inferred that the directional alignment of covalent groups in title pnictides may be triggered by the diamond-like sublattice trait of ionic motifs. Furthermore, by analyzing the coordination environment, we find that the coordination formations (A_4_Br) are still deeply related to the diamond-like structure. The (Sr_4_Br) and (Ba_4_Br) units are encapsulated by 24 neighbor P atoms, forming (Sr_4_Br)@P_24_ and (Ba_4_Br)@P_24_ polyhedrons, respectively (Fig. 3G). The (Ba_4_Br)@P_24_ polyhedrons are further interconnected by sharing 2 Ba atoms and 12 P atoms to form 1D infinite [Ba_6_Br_2_]@P_36_ jagged chains (Fig. 3I). In GaP, the (P_4_Ga_13_)@P_24_ cubic dodecahedron can be constructed by 13 (GaP_4_) tetrahedra centered on 1 GaP_4_ tetrahedron (Fig. 3H). In ZnGeP_2_, (Zn_5_Ge_8_P_4_)@P_24_ and [Ge_5_Zn_8_P_4_]@P_24_ cubic dodecahedron can also be built via five ZnP_4_ and eight GeP_4_ centered on one ZnP_4_ and five GeP_4_ and eight ZnP_4_ centered on one GeP_4_. The [Zn_5_Ge_8_P_4_]@P_24_ and [Ge_5_Zn_8_P_4_]@P_24_ are also interconnected by sharing 2 Zn atoms and 12 P atoms to form 1D infinite [Zn_11_Ge_13_P_8_]@P_36_ jagged chains (Fig. 3J) like those in BBISP. At a geometric shape, size, and coordination environment, the (Sr_4_Br)@P_24_ polyhedrons and [Ba_6_Br_2_]@P_36_ jagged chains are close to the (P_4_Ga_13_)@P_24_ cubic dodecahedron (8.13 Å versus 8.21 Å; 7.91 Å versus 8.21 Å) and [Zn_11_Ge_13_P_8_]@P_36_ jagged chains (8.20 Å versus 8.21 Å; 12.91 Å versus 13.40 Å), respectively, indicating that they have a similar electronic structure. It means that the electrostatic force interactions on neighboring covalent tetrahedron groups created by (Sr_4_Br) and (Ba_4_Br) ionic units are similar with corresponding local clusters in the diamond-like structure. The calculated electrostatic potential diagrams well confirm that (Sr_4_Br)@P_24_ and (Ba_6_Br_2_)@P_36_ have a very similar electronic structure and electrostatic field distribution with (P_4_Ga_13_)@P_24_ and [Zn_11_Ge_13_P_8_]@P_36_ (Fig. 4). As a result, the circumambient tetrahedron groups (Fig. 2, D and H) codirectionally arranged due to the diamond-like electrostatic force field effect of ionic motifs. Furthermore, the ^0D^[Sr_4_Br]^7+^ and [Ba3Br]5+∞1D ionic motifs are arrayed parallelly (fig. S4), actuating directional assembly of the global covalent tetrahedron groups (Fig. 2, B and F). Similar cases were also observed in salt-inclusion chalcogenides, e.g., [K_4_Cl][CdGa_9_S_16_] and [K_2_PbBr][Ga_7_S_12_], reported by Liu and colleagues (*26*–*28*).

### Optical properties

**Fig. 5.**
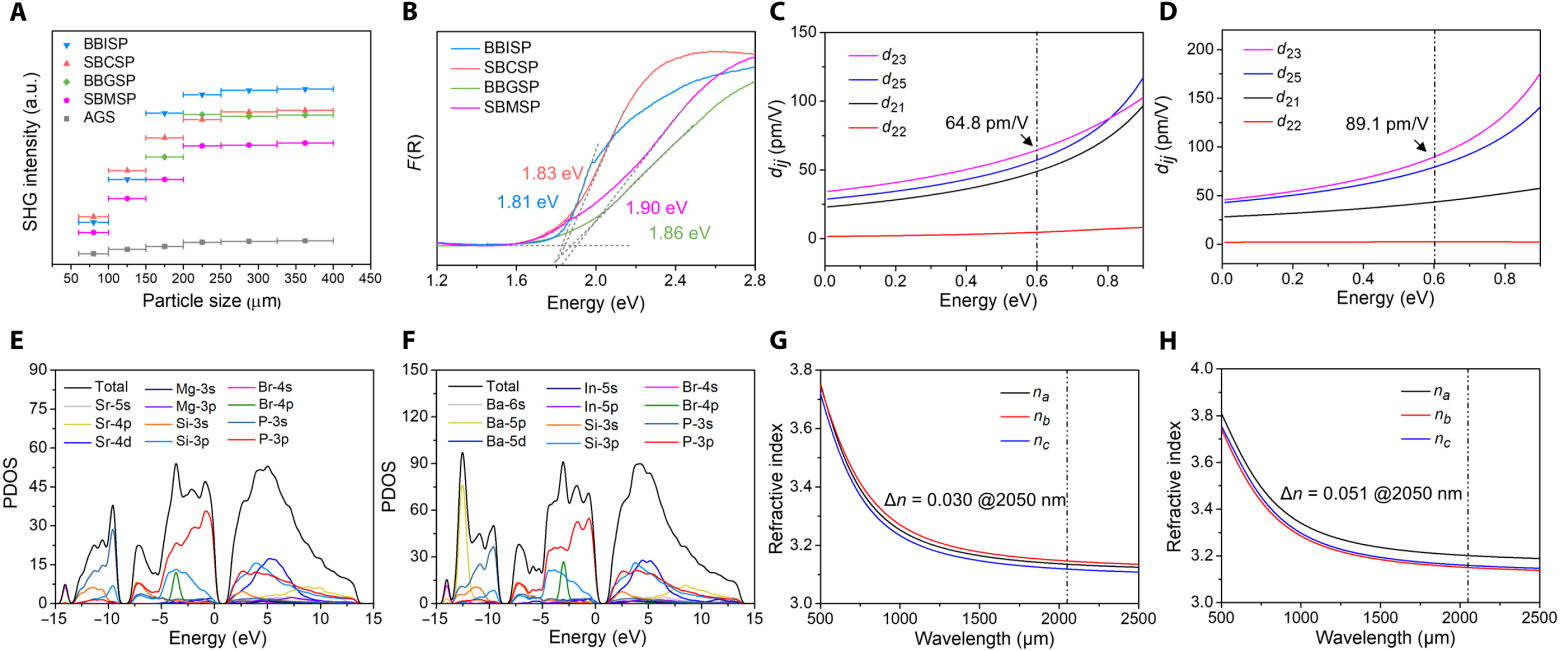
Optical properties and PDOS. (**A**) Particle size versus SHG response curves. a.u., arbitrary units. (**B**) Measured optical bandgaps. (**C** and **D**) Calculated NLO coefficients of SBMSP and BBISP. (**E** and **F**) Calculated PDOS of SBMSP and BBISP. (**G** and **H**) Calculated refractive index dispersion curves of SBMSP and BBISP.

The polycrystalline SHG responses were investigated through the Kurtz and Perry method under a 2.05-μm pump laser (*29*). SBMSP, SBCSP, BBGSP, and BBISP exhibited phase-matching SHG signals of 5.2 to 7.5 times that of AgGaS_2_ (AGS) (Fig. 5A), strongest in all reported A-M-Pn system and NLO crystals, e.g., BaGe_2_P_2_ (3.3 × AGS), BaSi_7_P_10_ (3.2 × AGS), SrIn_3_Si_4_P_9_ (0.12 × AGS), Ba_2_Si_3_P_6_ (1 × AGS), and other NLO pnictides (*30*–*35*). Under the restriction of Kleinman’s symmetry, SBMSP, SBCSP, BBGSP, and BBISP have four independent nonvanishing second-order susceptibility tensor components, namely, *d*_21_, *d*_25_, *d*_23_, and *d*_22_, which were calculated via the ABINIT software (Fig. 5, C and D; fig. S11, C and D; and table S6). The largest tensor component, *d*_23_, for SBMSP, SBCSP, BBGSP, and BBISP is 64.8, 76.2, 71.3, and 89.1 pm/V, respectively, accordant with the experimental SHG intensities. According to anionic group theory, the NLO performances depend on the superposition effect of second-order susceptibility of the microscopic groups. Their colossal NLO performances originate from the highly directional arrangement of the tetrahedron groups in covalent building blocks contributing to complete the superposition effect of microscopic second-order susceptibility (*36*). To quantitatively evaluate the arrangement level of tetrahedron groups along the largest NLO susceptibility component, *d*_23_, the structural criterion *C* of tetrahedron groups was calculated based on anionic group theory (*37*). The *C* value ranges from 0 to 1, which represents the most unfavorable and most favorable arrangement of the global tetrahedron groups, respectively. The results (Table 1) show that they all have a high *C* value of 0.982 to 0.984 comparable to ZnGeP_2_ (*C* = 0.999) and CdSiP_2_ (*C* = 0.982), indicating that group arrangements are highly uniform. This is favorable to geometric superposition of the microscopic second-order susceptibility of tetrahedron groups, leading to large second-order NLO performances. The experimental optical bandgaps based on the Kubelka-Munk function show that title crystals all have wide bandgaps ranging from 1.81 to 1.90 eV (Fig. 5B). This demonstrates that the bandgap of the A-M-Pn-X system with the introduction of halogens can be effectively increased (the related atomic mechanism is analyzed in the following content). The powder laser-induced damage threshold (LIDT) measurements show that the LIDT values of these four pnictides are eight to nine times that of AgGaS_2_ (table S7), larger than that of SrSi_7_P_10_ (~7 × AGS), BaSi_7_P_10_ (~7 × AGS), and SrIn_3_Si_4_P_9_ (~7 × AGS).

**Table 1.** Summary of key physical properties. DM, dipole moment.

	SBMSP	SBCSP	BBGSP	BBISP
SHG (× AGS)	5.2	6.6	6.4	7.5
*E*_g_ (eV)	1.90	1.83	1.86	1.81
Δ*n*	0.03	0.036	0.033	0.051
*d*_23_ (pm/V)	64.8	76.2	71.3	89.1
IR range (μm)	9.4	9.4	9.4	9.4
*C*	0.982	0.984	0.983	0.982
DM (D)	14.4	12.9	22.7	7.6

**Fig. 6.**
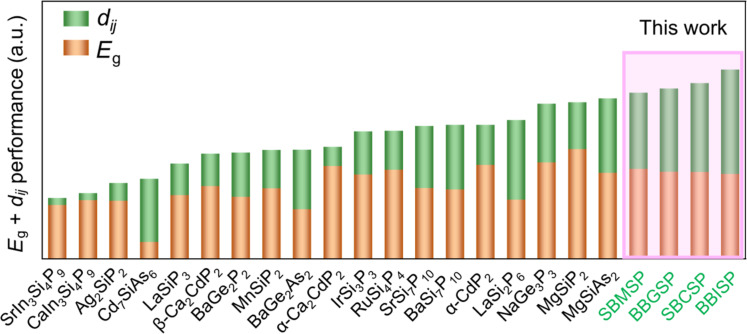
Comprehensive optical performances compared to all reported A-M-Pn and other NLO pnictides.

Compared to all reported A-M-Pn system and some other NLO pnictides, the title crystals show the best comprehensive optical performances defined as the weighted sum of bandgap and largest NLO coefficient component (Fig. 6) (*38*–*45*). IR transmittance spectra (fig. S5) show that title crystals all have no obvious absorption peaks until 1067 cm^−1^ (9.4 μm), comparable to the IR transparency cutoffs of other silicon-phosphides, CdSiP_2_ (~9 μm), Ag_2_SiP_2_ (~10 μm), and MgSiP_2_ (10.3 μm), because their IR cutoffs are all determined by two-phonon absorption of the Si-P bond vibration.

### Structure-property relationship

To better understand the structure-property relationship, first-principles calculations were performed. SBMSP, SBCSP, BBGSP, and BBISP have direct calculated bandgaps of 1.32, 1.28, 1.31, and 1.26 eV (fig. S9), respectively, well consistent with the order of the experimental values (1.90, 1.83, 1.86, and 1.81 eV). The calculated partial density of states (PDOS) (Fig. 5, E and F, and fig. S10) show that the top of the valence band (VB) was dominated by P-3p and Si-3p orbitals and that the bottom of the conduction band (CB) was mainly composed of P-3p and Si-3p orbitals mixed with some Ba-5d/Sr-4d and Si-3s orbitals. Noting that Ba-5d/Sr-4d empty orbitals make a contribution to the VB top indicates that the nonbonding electrons on the P atom are delocalized between the long-range interaction of Ba 5d/Sr-4d and P 3p, resulting in a lower electron transition energy from P 3p to Ba 5d/Sr-4d. This phenomenon is more prominent in the A-M-Pn system, and even the A-P interaction dominates the edge of the bandgap. In that case, the A-P interaction is not pure ionicity anymore but “intermetallic” with more delocalized electron distribution, pulling the CB edge to a lower energy level. Thus, pnictides of the A-M-Pn system usually do not have wide bandgaps, while in the A-M-Pn-X system, halogens with strong electronegativity have lower orbital energy at the VB top (Br 4p ranges from −2 to −5 eV), which pull down the energy level of the VB edge. Moreover, the A-Br interaction in the A_4_Br unit effectively weakens the CB top contribution of Ba 5d/Sr 4d. Therefore, the bandgap of title crystals is improved expectedly. The calculated refractive index dispersion curves show that the title crystals have birefringence of 0.030, 0.036, 0.033, and 0.051 at 2.05-μm laser irradiation (Fig. 5, G and H, and figs. S11, A and B, and S12), accordant with the phase-matching behavior of experimental SHG intensities.

**Fig. 7.**
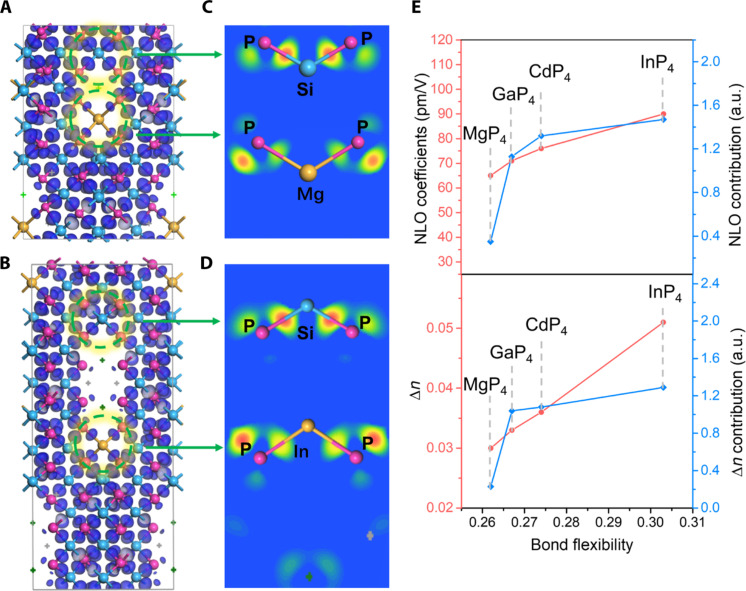
EDD and bond flexibility–optical property relations. (**A** and **B**) EDD isosurface distribution of SBMSP and BBISP. (**C** and **D**) Slice EDD field distribution of SBMSP and BBISP along Si-P and M2-P bonds. (**E**) Bond flexibility versus NLO performances and birefringence.

The electron density difference (EDD) analyses show that each middle of the Si-P bond within the SiP_4_ tetrahedron has a remarkable attractor (Fig. 7, A to D, and fig. S13), indicating that the Si-P bonds are strong covalent bonds. For M2P_4_ groups, MgP_4_, CdP_4_, GaP_4_, and InP_4_ tetrahedra, the bonds between M and P atoms also exhibit weaker attractors shifting to P, which implies that these M2-P bonds are more polar and flexible. To quantitatively evaluate the characteristics of these covalent bonds, the Mulliken bond population and bond flexibility index are calculated. The bond population of the Si-P, In-P, Ga-P, Mg-P, and Cd-P bond is 0.68, 0.56, 0.53, 0.45, and 0.39 (table S9), indicating that their covalent nature decreases gradually. The 3D skeletons constructed by SiP_4_ tetrahedra with high bonding rigidity and strength give these four crystals excellent physicochemical stability. Thermogravimetric (TG)/differential thermal analysis (DTA) curves show that they can stabilize up to 815°~853°C (fig. S6) without decomposition, more robust than most pnictides and chalcogenides. After decomposition temperature, the main decomposition products are MgSiP_2_ for [Sr_4_Br]_2_[Mg_3_Si_25_P_40_], CdSiP_2_ for [Sr_4_Br]_2_[Cd_3_Si_25_P_40_], GaP for [Ba_3_Br][GaSi_10_P_16_], and InP for [Ba_3_Br][InSi_10_P_16_] (fig. S7). In addition, they are not sensitive to air humidity and have no obvious change for 6 months under ambient conditions (fig. S8).

The bond flexibility index (*F*) calculations show that Si-P bonds have *F* values of 0.266 to 0.268, comparable to Ga-P (0.267) bonds, larger than Mg-P (0.262), and lower than In-P (0.303) and Cd-P bonds (0.274). The NLO performances originate form geometrical superposition of microscopic second-order susceptibility of the groups, and the microscopic second-order susceptibility depends on the bond flexibility resulting from the flexibility of the electronic motion in a chemical bond subjected to the perturbation of the external optical electric field (*46*). Because these four crystals with SiP_4_ tetrahedra as the basic skeleton all have close group arrangement levels (*C* = 0.982~0.984) and same molar ratio of elements Si and P (5:8), the superposition effect of groups is approximately identical. However, their NLO properties exhibit a significant difference (*d*_23_ ranges from 65 to 90 pm/V). This intriguing phenomenon may be due to various degrees of synergistic effects caused by secondary building blocks (M2P_4_ tetrahedra) with distinct bond flexibility. As shown in Fig. 7E, the NLO coefficients are vitally related with the bond flexibility of M2P_4_. The higher the flexibility of the M2-P bond, the greater the NLO contribution of the M2P_4_ group and the larger the NLO coefficient. Moreover, the bond flexibility of the M2P_4_ group also makes a similar influence on birefringence. The secondary building blocks play a key role in regulating NLO performances and birefringence, although their stoichiometric proportion in the structure is very low.

## DISCUSSION

We designed and synthesized the first series of NCS A-M-Pn-X system pnictides: [Sr_4_Br]_2_[M^II^_3_Si_25_P_40_] (M^II^ = Mg, Cd) and [Ba_3_Br][M^III^Si_10_P_16_] (M^III^ = Ga, In). They exhibit strong SHG effects (5.2 to 7.5 × AGS), suitable bandgaps (1.81 to 1.90 eV), moderate birefringence (0.030 to 0.051), and wide IR transparent ranges. Structure mechanism analysis uncover that the parallel ^0D^[Sr_4_Br]^7+^ and [Ba3Br]5+∞1D ionic arrays with diamond-like topology sublattice and local coordination geometry drive the codirectional arrangement of covalent tetrahedron groups. Moreover, the chemical bond analysis confirms that secondary covalent building blocks (M2P_4_ tetrahedra) with distinct bond flexibility can achieve the dedicated regulation of the NLO properties and birefringence. This work develops a previously unknown research system of inorganic pnictides and provides an approach for efficiently designing NCS crystalline materials.

## MATERIALS AND METHODS

### Materials and syntheses

The raw materials of Mg (3N), Cd (3N), Si (3N), P (5N), In (3N), Li (3N), SrBr_2_ (3N), and BaBr_2_ (3N) were used as received without further purification. The charge rate of the component element is Mg:Si:P:SrBr_2_ = 0.5:3:5:2 for SBMSP, Cd:Si:P:SrBr_2_ = 0.5:3:5:2 for SBCSP, Ga:Si:P:BaBr_2_ = 1:3:5:4 for BBGSP, and In:Si:P:BaBr_2_ = 1:3:5:4 for BBISP. The mixtures were thoroughly ground and mixed in a glove box under Ar atmosphere and then loaded in fused silica tubes under a vacuum of 10^−3^ Pa. Subsequently, the tube was placed in a muffle furnace and heated to 1000°C, holding for 3 days, slowly cooled to 800°C at a rate of 3°C/hour, and lastly cooled to 300°C at a rate of 10°C/hour before naturally cooling. The air-stable and moisture-stable red polycrystalline block of title compounds was obtained and then washed using deionized water to remove the SrBr_2_ and BaBr_2_ flux.

### Single-crystal X-ray diffraction

The diffraction data of SBMSP and BBISP were collected on a Bruker SMART APEX III 4K charge-coupled device diffractometer with Mo Kα radiation (λ = 0.71073 Å) at 293 (2) K. The data were integrated by APEX III, and the multiscan method was used to the absorption corrections. The diffraction data of SBCSP and BBISP were collected on a Rigaku XtaLAB Pro MM003 Cu/Mo diffractometer with Mo Kα radiation (λ = 0.71073 Å) and Cu Kα radiation (λ = 1.54178 Å) at 293 (2) K. The data were integrated by the CrysAlisPro program, and the multiscan method was used to the absorption corrections. The crystal structures of these four compounds were solved by the SHELXT intrinsic phasing methods and refined with anisotropic thermal parameters for all atoms by SHELXL full-matrix least-squares fitting on *F*^2^ on the Olex2 program (*47*, *48*). The PLATON program was used to check the correctness of the structures, and no higher symmetries were found (*49*). The crystal data and structure refinement parameters were shown in table S1. Some structural parameters including interatomic distances, final refined atomic positions, and anisotropic displacement parameters are listed in tables S2, S3, and S4, respectively.

### Powder X-ray diffraction

The PXRD pattern data of title compounds were collected from 10° to 70° (2θ) with a step width size of 0.01° and a step time of 2 s on a SmartLab9KW powder x-ray diffractometer with Cu Kα radiation. The PXRD patterns were performed Rietveld refinement using GSAS-II.

### Elemental analysis

The elemental composition analysis was performed on a field-emission scanning electron microscope (Quanta FEG 250) equipped with an energy-dispersive x-ray spectrometer.

### Optical properties

The Shimadzu SolidSpec-3700DUV spectrophotometer was used to measure the UV-vis-NIR diffuse reflectance spectrum of title compounds in a wavelength range from 240 to 2400 nm at room temperature. BaSO_4_ was selected as the standard for 100% reflectance comparison. The reflectance value is converted to absorbance by using the Kubelka-Munk function (*50*). The IR transmittance spectra were recorded on a Frontier Mid-IR FTIR/STA6000-TL9000-Clarus SQ8 spectrophotometer in the range of 4000 to 400 cm^−1^. Powder samples and dry KBr were mixed and ground into fine powder and then were pressed into transparent sheets for the measurements.

### Thermal stability analysis

TG and DTA were performed on a NETZSCH STA 449F3 unit under N_2_ atmosphere at a 10°C/min heating rate. An amount of 20 mg of title compounds was ground into fine powder and enclosed in an Al_2_O_3_ crucible. The well-prepared samples were heated from room temperature to 1200°C at a rate of 10°C/min.

### Powder SHG measurements

Polycrystalline SHG responses were measured with the Kurtz-Perry method using a Q-switched Nd–yttrium-aluminum-garnet solid-state laser with a wavelength of 2050 nm (*29*). Polycrystalline samples were ground and sieved into several distinct particle size ranges of 60 to 100, 100 to 150, 150 to 200, 200 to 250, 250 to 325, 325 to 400, and 400 to 500 μm and then pressed into the container with a thickness of 1 mm and diameter of 8 mm. Polycrystalline AgGaS_2_ was prepared with the same size range as the comparison reference.

### Powder LIDT measurements

The LIDTs at a particle size range of 150 to 200 μm was measured through a single-pulse measurement method with AgGaS_2_ as the reference. The samples were loaded into the container same as polycrystalline SHG response measurements use. A power-tunable 1064-nm laser with a pulse width τ of 10 ns and operating frequency of 1 Hz was used to radiate the sample surface until the fixed size damage spot (0.1 cm^2^) occurs. The laser energy *E* was recorded. The LIDT value was calculate with the equation *I*(threshold) = *E*/(π*r*^2^τ).

### Theoretical calculation details

The electronic structure calculations were performed by the first-principles calculations in the CASTEP package based on density functional theory, with the norm-conserving pseudopotentials (*51*–*57*). The Perdew-Burke-Ernzerhof functional within the generalized gradient approximation (GGA) was applied for the exchange-correlation potential (*58*, *59*). The kinetic energy cutoff is set to be 990 eV. The Monkhorst-Pack grid size for self-consistent field calculation is 1 × 1 × 2, and the number of k-points is 1 for the Brillouin zone (*60*, *61*). Because of the discontinuity of exchange correlation, bandgaps calculated by the GGA method are usually smaller than experimental values, so a scissor operator was adopted to raise the CBs to match the experimental value for optical property calculation. The dynamic second-order nonlinear susceptibilities χ*_ijk_* (2ω, ω, ω) were calculated using the ABINIT software based on the density functional perturbation theory; the response calculation is at the level of the independent particle approximation (*62*).
