# Supplementary material for: Achieving strong optical nonlinearity and wide bandgap of pnictides via ionic motif–driven directed assembly of covalent groups
Source: Sci Adv. 2024 Nov 13;10(46):eadr2389. doi: 10.1126/sciadv.adr2389 (PMC11559624; doi:10.1126/sciadv.adr2389)
Supplement: Supplementary file 1 — Figs. S1 to S13 Tables S1 to S9 Legends for data S1 to S4 [file sciadv.adr2389_sm.pdf]

Science Advances
AAAS

Supplementary Materials for

Achieving strong optical nonlinearity and wide bandgap of pnictides via ionic motif–driven directed assembly of covalent groups

Lihua Gao *et al.*

Corresponding author: Jindong Chen, cjd1225@email.tjut.edu.cn; Ning Ye, nye@email.tjut.edu.cn

Sci. Adv. **10**, eadr2389 (2024)
DOI: 10.1126/sciadv.adr2389

The PDF file includes:

Figs. S1 to S13
Tables S1 to S9
Legends for data S1 to S4

Other Supplementary Material for this manuscript includes the following:

Data S1 to S4

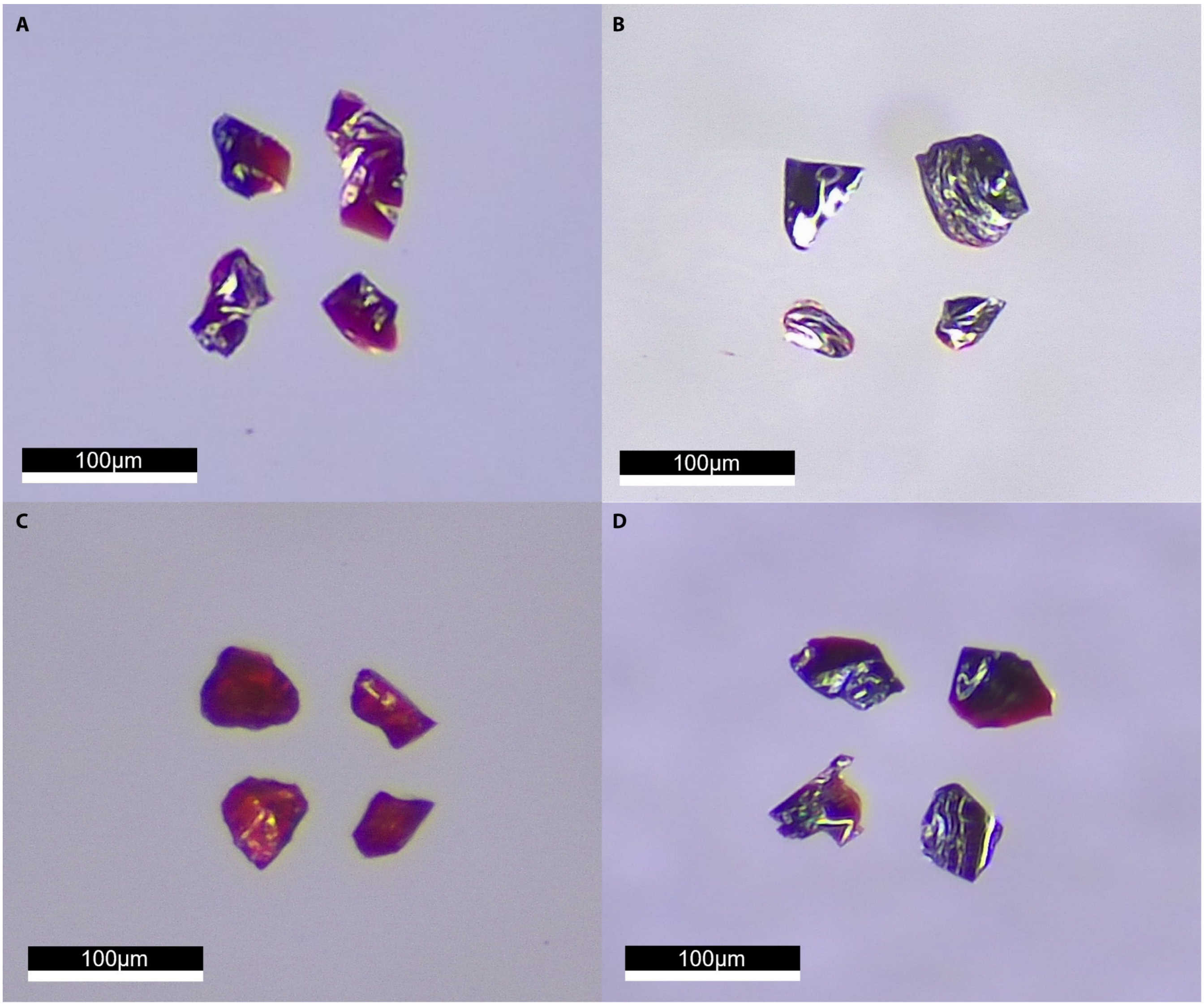

Fig. S1. Crystal photomicrographs. (A to D) Crystal photomicrographs of SBMSP, SBCSP, BBGSP and BBISP.

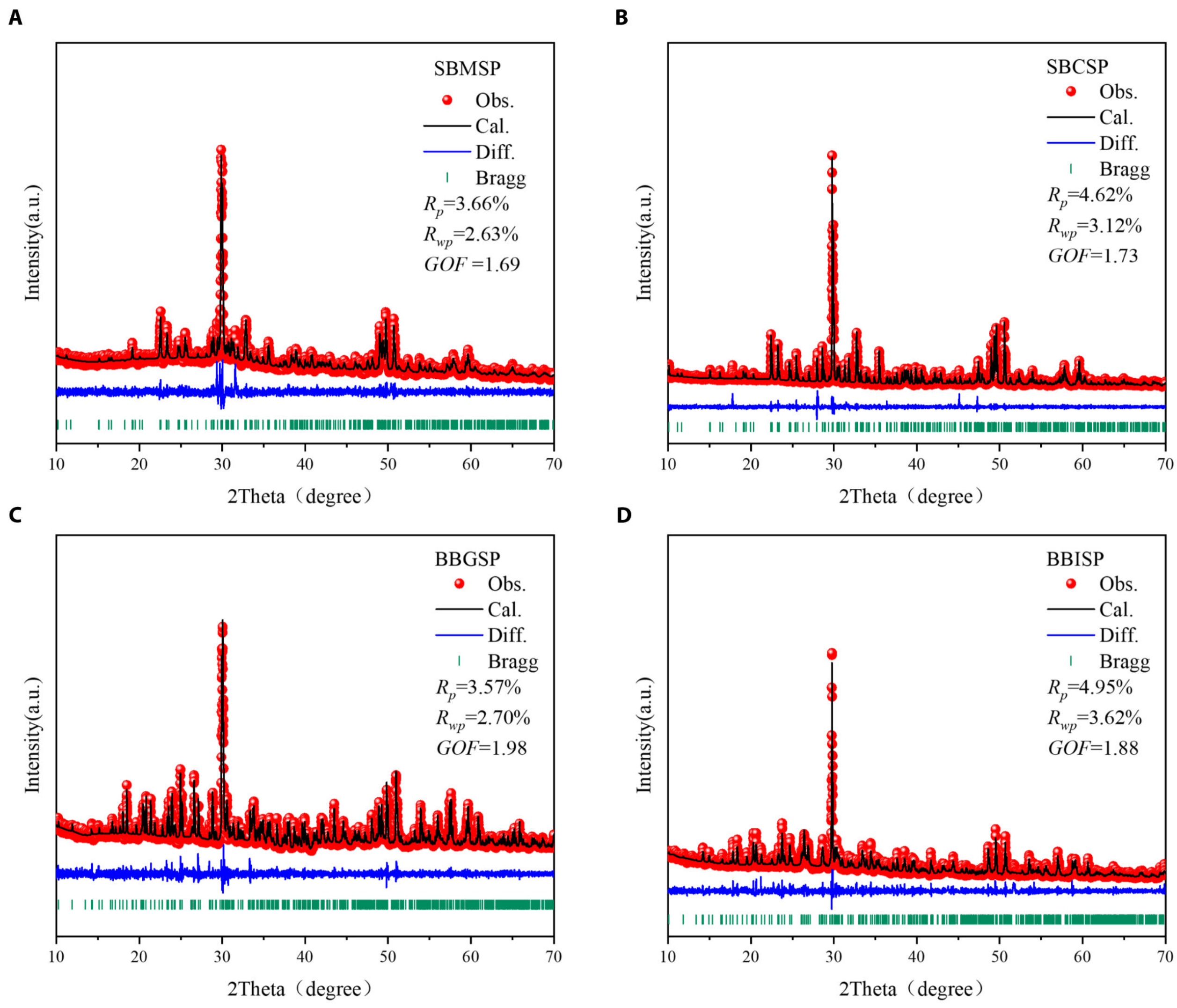

Fig. S2. Powder XRD Rietveld refinement patterns. (A to D) Powder XRD Rietveld refinement patterns SBMSP, SBCSP, BBGSP and BBISP.

A

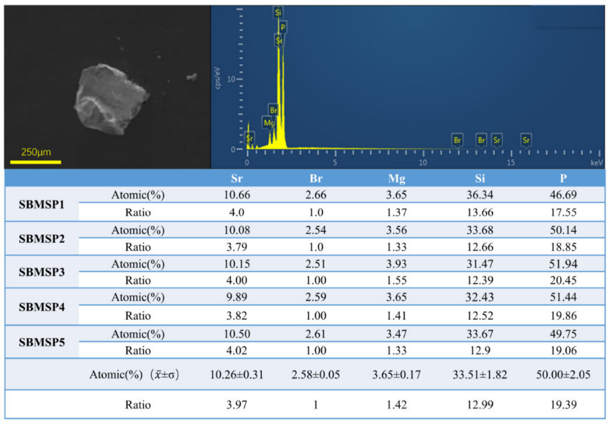

		Sr	Br	Mg	Si	P
SBMSP1	Atomic(%)	10.66	2.66	3.65	36.34	46.69
	Ratio	4.0	1.0	1.37	13.66	17.55
SBMSP2	Atomic(%)	10.08	2.54	3.56	33.68	50.14
	Ratio	3.79	1.0	1.33	12.66	18.85
SBMSP3	Atomic(%)	10.15	2.51	3.93	31.47	51.94
	Ratio	4.00	1.00	1.55	12.39	20.45
SBMSP4	Atomic(%)	9.89	2.59	3.65	32.43	51.44
	Ratio	3.82	1.00	1.41	12.52	19.86
SBMSP5	Atomic(%)	10.50	2.61	3.47	33.67	49.75
	Ratio	4.02	1.00	1.33	12.9	19.06
	Atomic(%) $(\bar{x}\pm\sigma)$	10.26±0.31	2.58±0.05	3.65±0.17	33.51±1.82	50.00±2.05
	Ratio	3.97	1	1.42	12.99	19.39

B

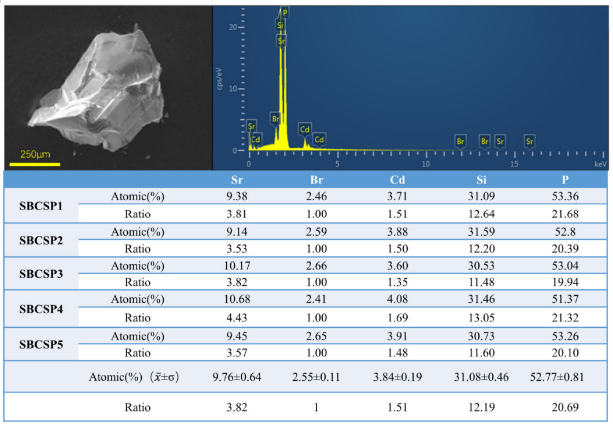

		Sr	Br	Cd	Si	P
SBCSP1	Atomic(%)	9.38	2.46	3.71	31.09	53.36
	Ratio	3.81	1.00	1.51	12.64	21.68
SBCSP2	Atomic(%)	9.14	2.59	3.88	31.59	52.8
	Ratio	3.53	1.00	1.50	12.20	20.39
SBCSP3	Atomic(%)	10.17	2.66	3.60	30.53	53.04
	Ratio	3.82	1.00	1.35	11.48	19.94
SBCSP4	Atomic(%)	10.68	2.41	4.08	31.46	51.37
	Ratio	4.43	1.00	1.69	13.05	21.32
SBCSP5	Atomic(%)	9.45	2.65	3.91	30.73	53.26
	Ratio	3.57	1.00	1.48	11.60	20.10
	Atomic(%) $(\bar{x}\pm\sigma)$	9.76±0.64	2.55±0.11	3.84±0.19	31.08±0.46	52.77±0.81
	Ratio	3.82	1	1.51	12.19	20.69

C

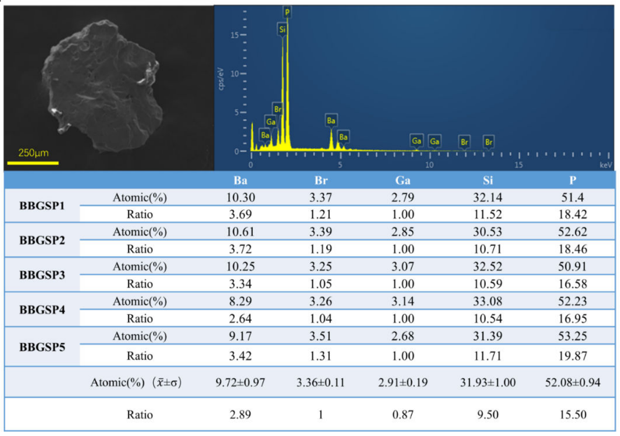

		Ba	Br	Ga	Si	P
BBGSP1	Atomic(%)	10.30	3.37	2.79	32.14	51.4
	Ratio	3.69	1.21	1.00	11.52	18.42
BBGSP2	Atomic(%)	10.61	3.39	2.85	30.53	52.62
	Ratio	3.72	1.19	1.00	10.71	18.46
BBGSP3	Atomic(%)	10.25	3.25	3.07	32.52	50.91
	Ratio	3.34	1.05	1.00	10.59	16.58
BBGSP4	Atomic(%)	8.29	3.26	3.14	33.08	52.23
	Ratio	2.64	1.04	1.00	10.54	16.95
BBGSP5	Atomic(%)	9.17	3.51	2.68	31.39	53.25
	Ratio	3.42	1.31	1.00	11.71	19.87
	Atomic(%) $(\bar{x}\pm\sigma)$	9.72±0.97	3.36±0.11	2.91±0.19	31.93±1.00	52.08±0.94
	Ratio	2.89	1	0.87	9.50	15.50

D

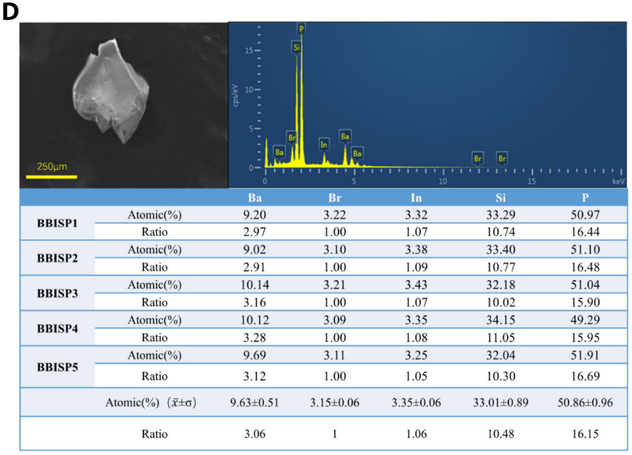

		Ba	Br	In	Si	P
BBISP1	Atomic(%)	9.20	3.22	3.32	33.29	50.97
	Ratio	2.97	1.00	1.07	10.74	16.44
BBISP2	Atomic(%)	9.02	3.10	3.38	33.40	51.10
	Ratio	2.91	1.00	1.09	10.77	16.48
BBISP3	Atomic(%)	10.14	3.21	3.43	32.18	51.04
	Ratio	3.16	1.00	1.07	10.02	15.90
BBISP4	Atomic(%)	10.12	3.09	3.35	34.15	49.29
	Ratio	3.28	1.00	1.08	11.05	15.95
BBISP5	Atomic(%)	9.69	3.11	3.25	32.04	51.91
	Ratio	3.12	1.00	1.05	10.30	16.69
	Atomic(%) $(\bar{x}\pm\sigma)$	9.63±0.51	3.15±0.06	3.35±0.06	33.01±0.89	50.86±0.96
	Ratio	3.06	1	1.06	10.48	16.15

Fig. S3. Energy-dispersive X-ray spectroscopy (EDS) analysis. (A to D) Microscopic image, EDS spectrum and five sets of element atomic ratio data SBMSP, SBCSP, BBGSP and BBISP.

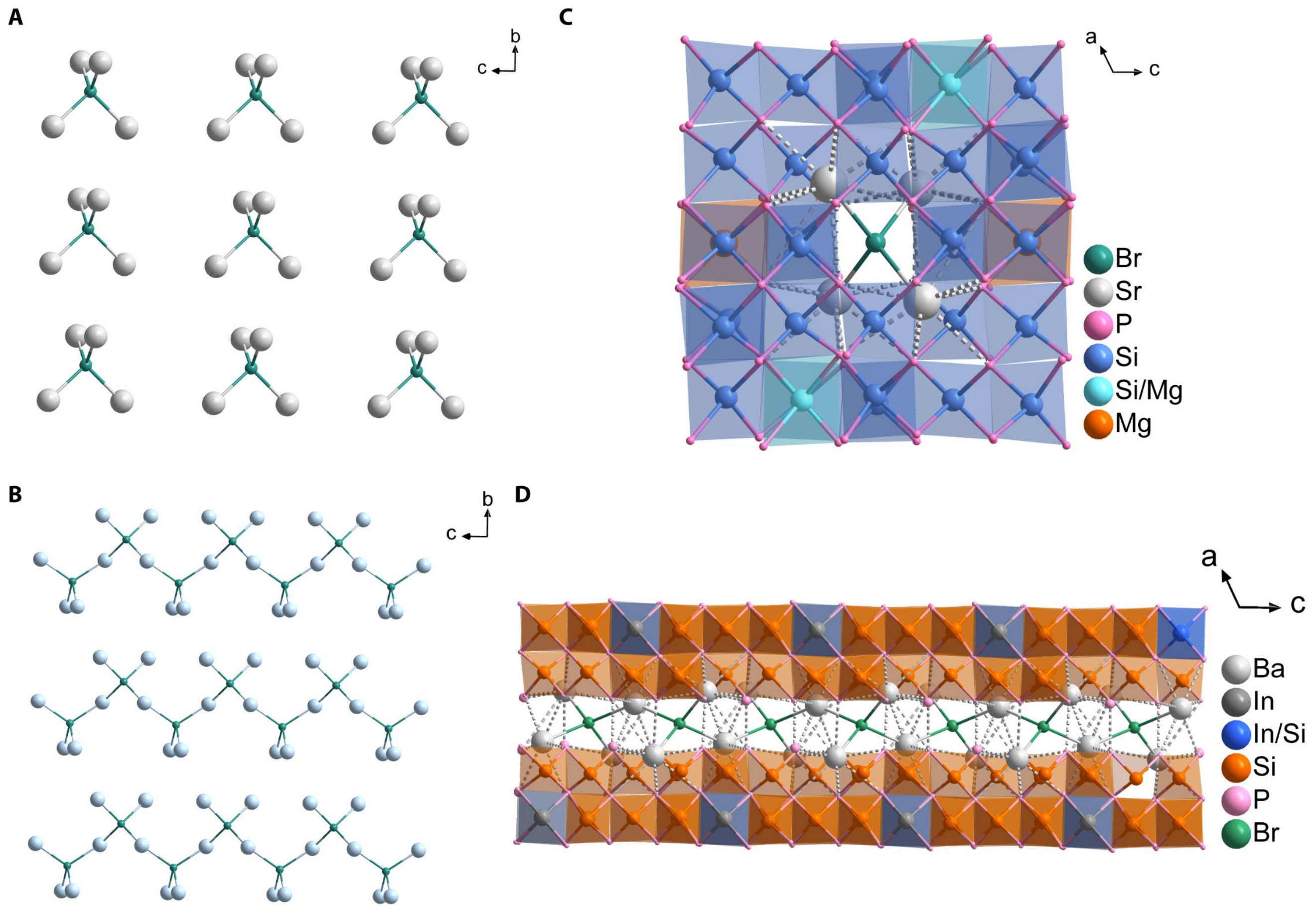

Fig. S4. Ionic motifs-driven directed dssembly of Covalent Groups. (A) The $^{0D}[Sr_4Br]^{7+}$ ionic motifs arrays in SBMSP and SBCSP. (B) $^{1D}_{\infty}[Ba_3Br]^{5+}$ ionic motifs in BBGSP and BBISP. (C, D) The circumambient Covalent tetrahedron groups of $^{0D}[Sr_4Br]^{7+}$ and $^{1D}_{\infty}[Ba_3Br]^{5+}$ ionic motifs.

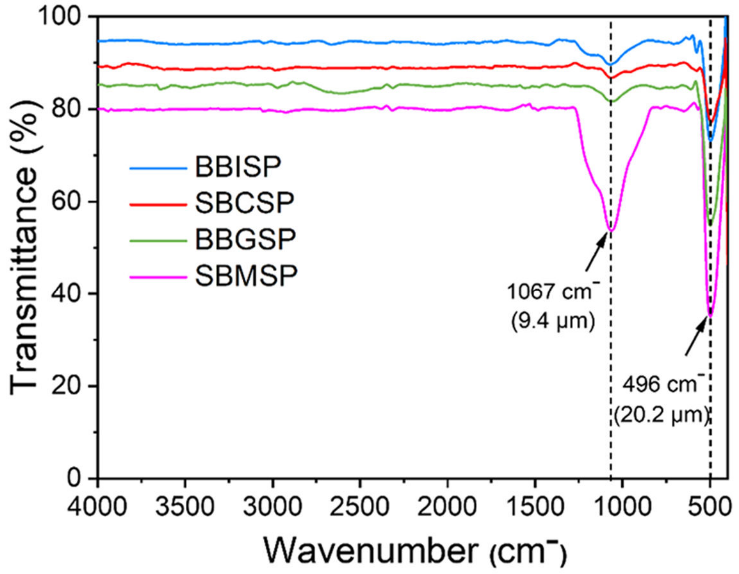

Fig. S5. IR transmittance spectra. IR transmittance spectra of SBMSP, SBCSP, BBGSP and BBISP.

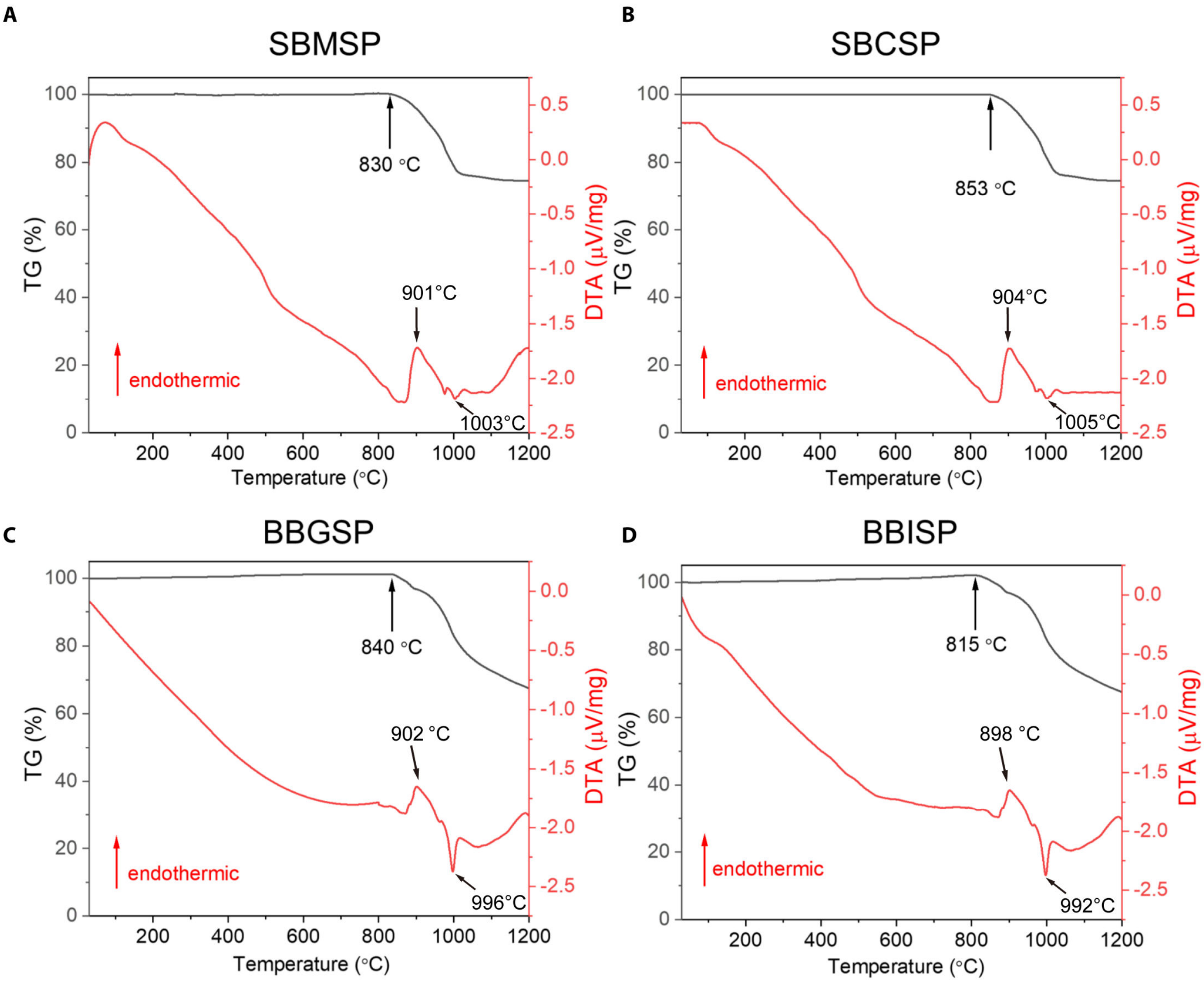

Fig. S6. TG/DTA analysis. (A to D) TG/DTA curves of SBMSP, SBCSP, BBGSP and BBISP.

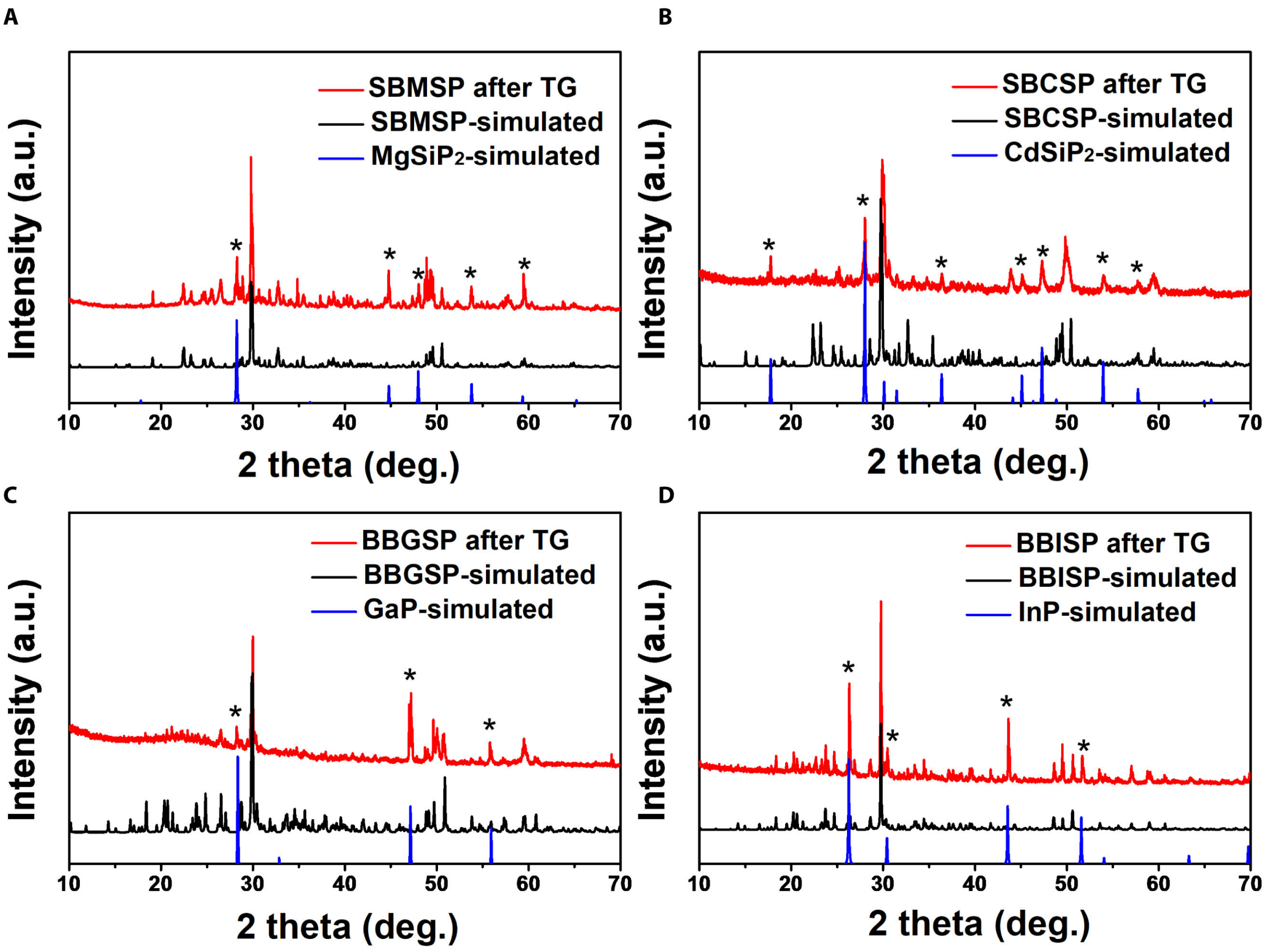

Fig. S7. Thermal decomposition product analysis. (A to D) Powder XRD patterns of SBMSP, SBCSP, BBGSP and BBISP after TG/DTA.

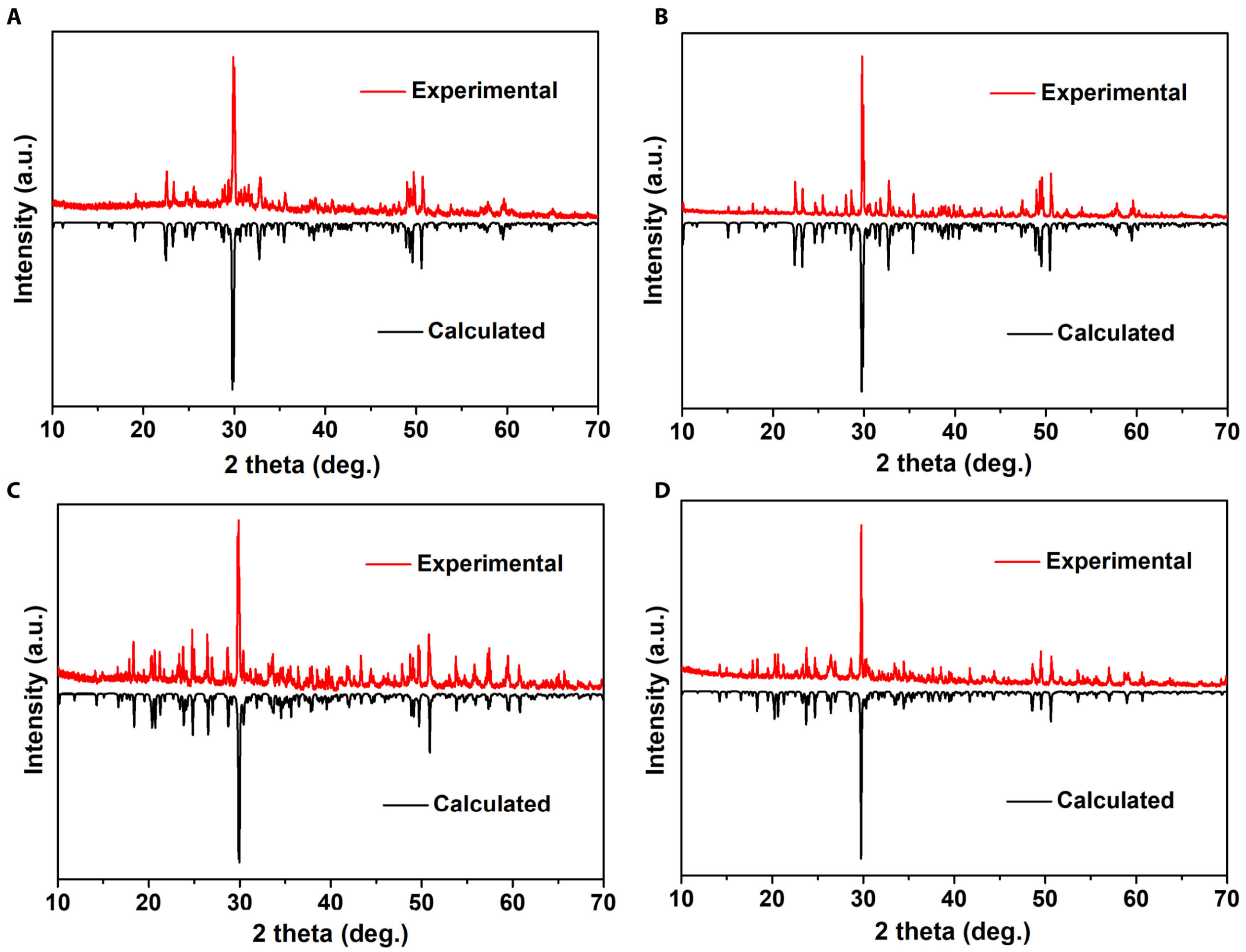

Fig. S8. Air-humidity stability verification. (A to D) Powder XRD patterns of SBMSP, SBCSP, BBGSP and BBISP ambiently exposed for six months.

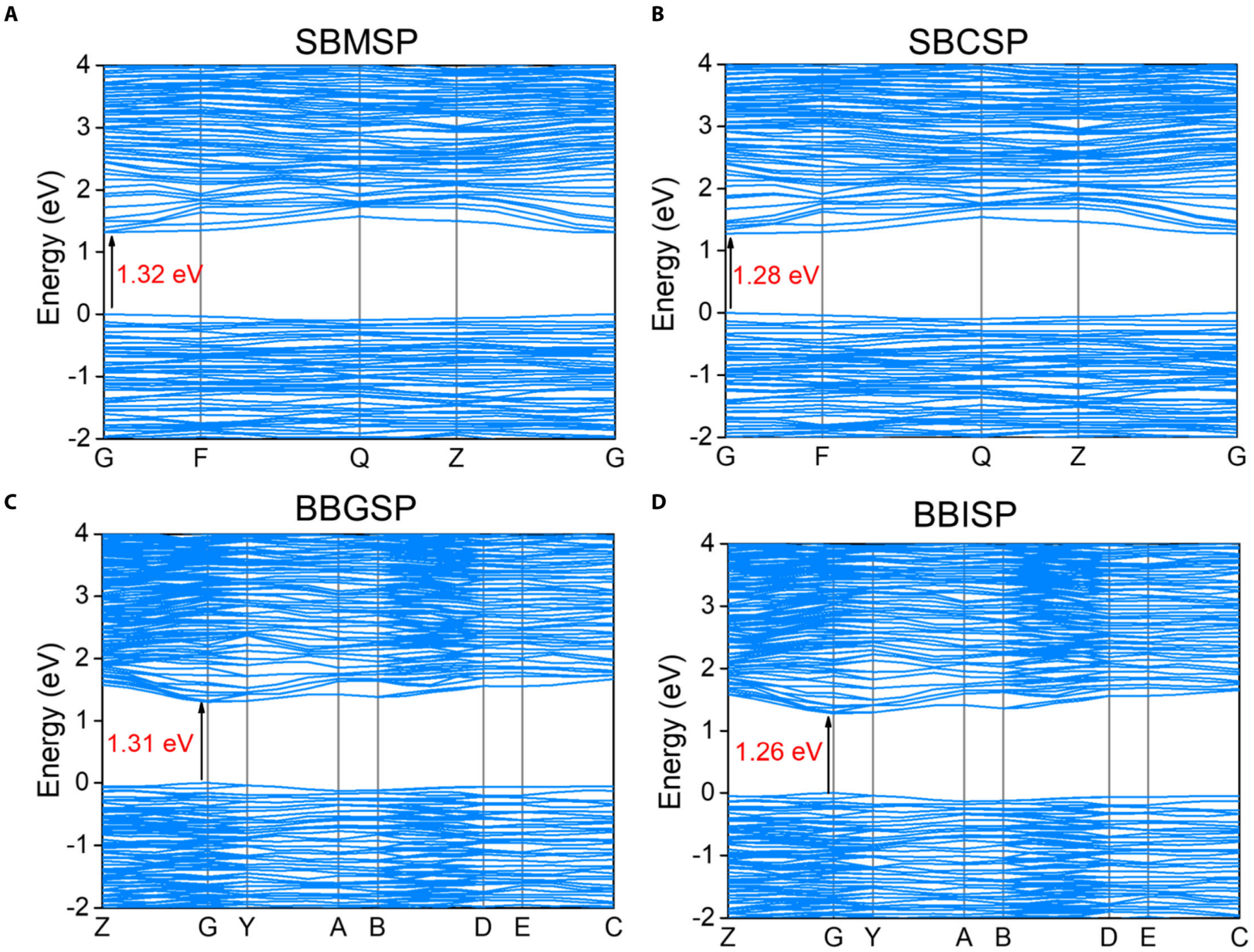

Fig. S9. Calculated electronic band structure. (A to D) Calculated electronic band structure of SBMSP, SBCSP, BBGSP and BBISP.

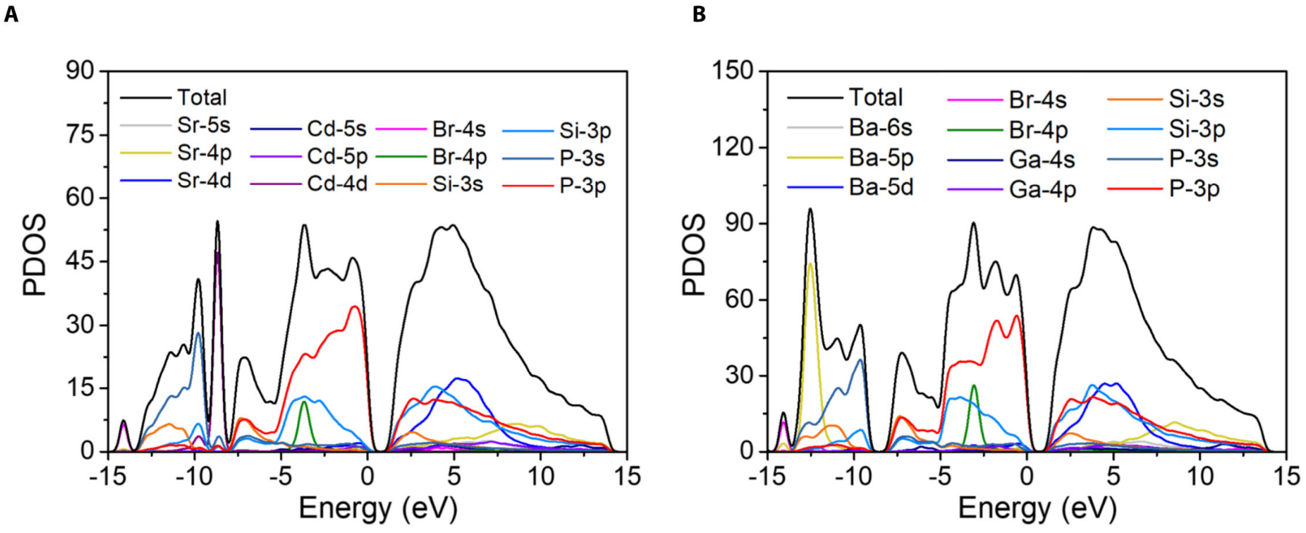

Figure S10. Calculated partial density of states (PDOS). (A, B) Calculated PDOS of SBCSP and BBGSP.

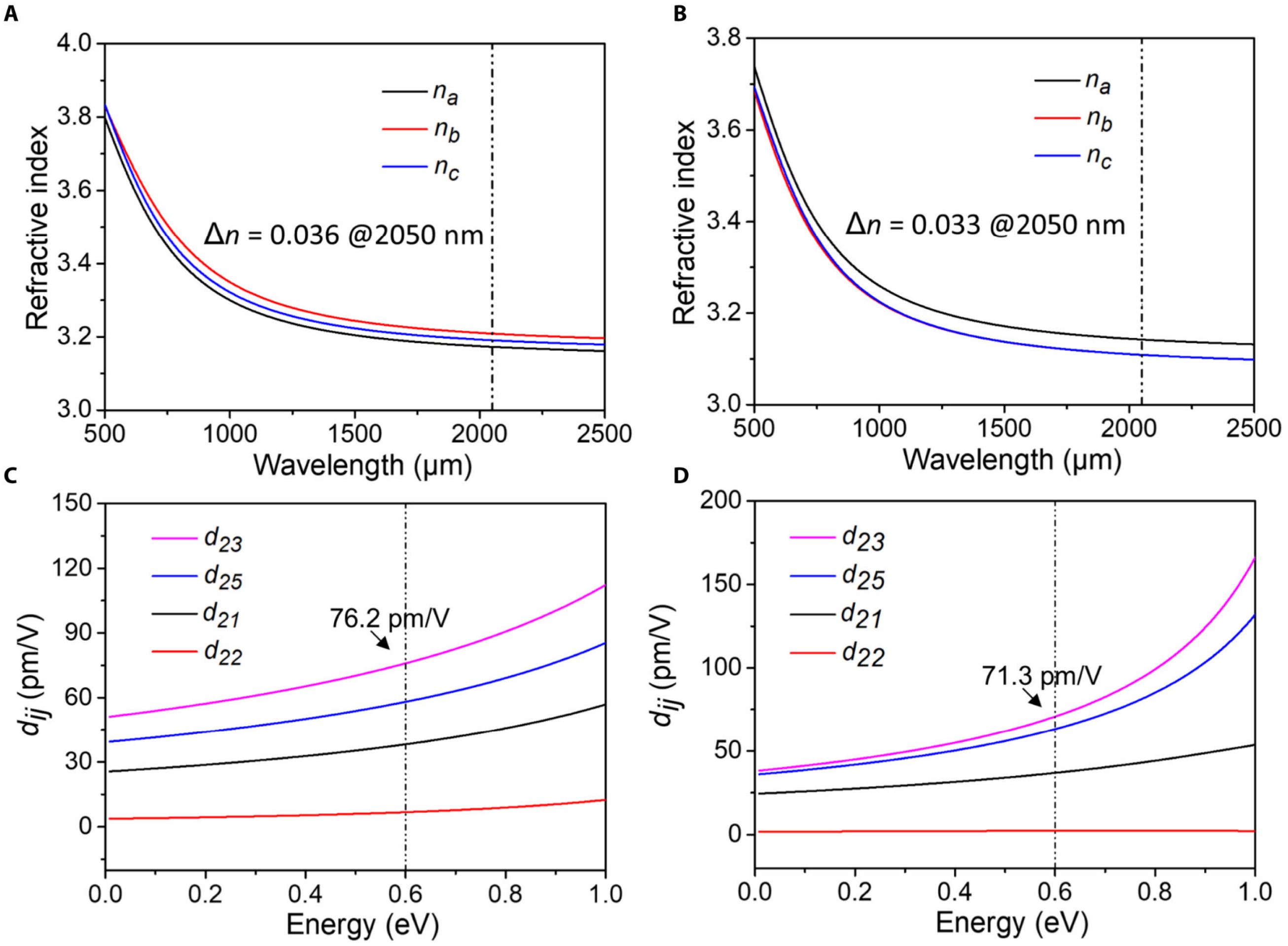

Fig. S11. Calculated NLO coefficients and refractive index dispersion curves. (A, B) NLO coefficients of SBCSP and BBGSP. (C, D) Refractive index dispersion curves of SBCSP and BBGSP.

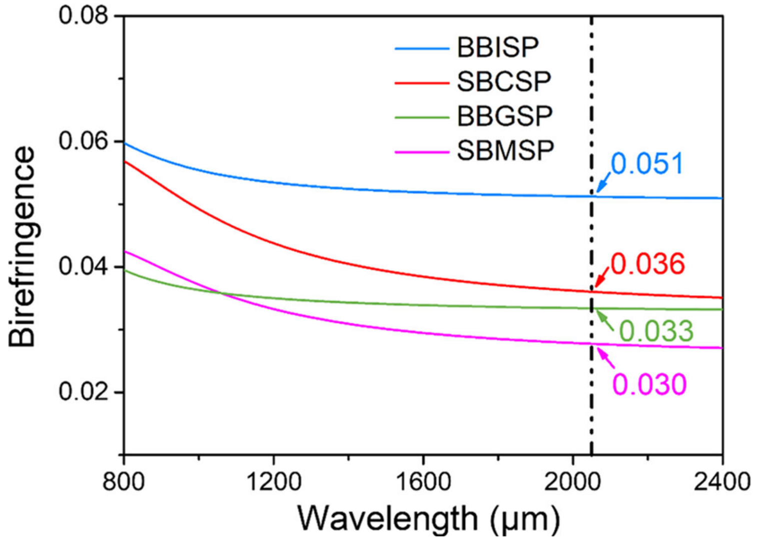

Fig. S12. Calculated Birefringence. Calculated Birefringence of SBMSP, SBCSP, BBGSP and BBISP.

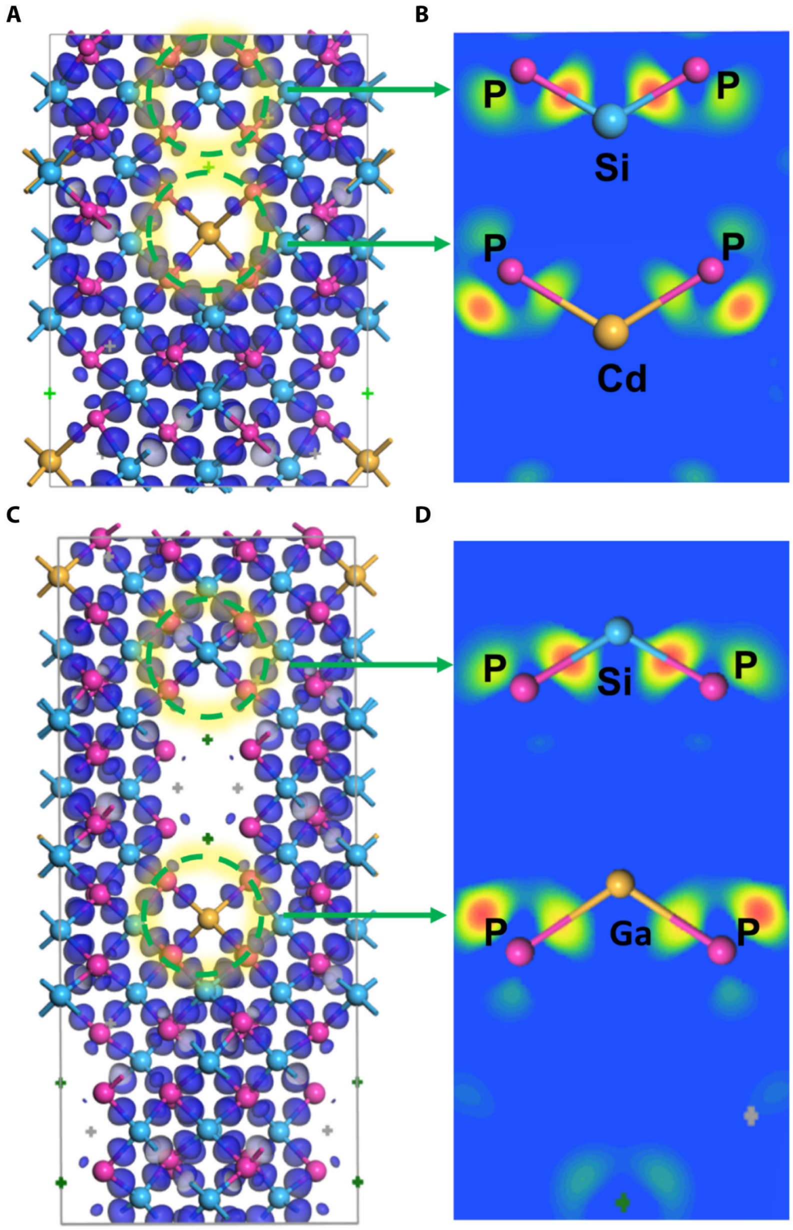

Fig. S13. Electron density difference patterns. (A, B) Electron density difference (EDD) iso-surface distribution of SBCSP and BBGSP. (C, D) Slice EDD field distribution of SBCSP and BBGSP along Si-P and M2-P bonds.

Table S1. Crystal data and structure refinement for title compounds.

Formula	SBMSP	SBCSP	BBGSP	BBISP
CCDC Numbers	2349072	2349073	2349074	2349075
Formula weight (amu)	1437.38	1569.51	1338.07	1383.17
Temp.(K)	293(2)	293(2)	293(2)	293(2)
λ(Å)	0.71073	0.71073	1.54178	0.71073
Crystal system	*monoclinic*	*monoclinic*	*monoclinic*	*monoclinic*
Space group	C2	C2	C2	C2
a (Å)	11.9085(3)	11.905(2)	11.8942(3)	11.9982(16)
b (Å)	15.1627(4)	15.2141(17)	25.0228(5)	25.042(3)
c (Å)	10.2942(4)	10.3085(14)	10.2739(3)	10.3782(14)
α (deg)	90	90	90	90
β (deg)	116.0480(10)	116.101(17)	115.290(3)	115.538(4)
γ (deg)	90	90	90	90
V (Å^3)	1669.97(9)	1676.8(5)	2764.71(14)	2813.6(6)
Z	2	2	4	4
ρcalc (g/cm3)	2.859	3.109	3.215	3.265
μ (mm^{-1})	8.997	9.858	48.579	7.711
F (000)	1360.0	1468	2456	2528.0
Reflections collected	10319	7932	9180	14650
Rint	0.0474	0.0357	0.0714	0.0403
R_1 (I >2σ(I))	0.0300	0.0219	0.0543	0.0489
wR$_2$ (I >2σ(I))	0.0542	0.0444	0.1370	0.1127
R_1 (all date)	0.0402	0.0251	0.0555	0.0583
wR_2 (all date)	0.0583	0.0455	0.1383	0.1182
Flack parameter	0.019(7)	0.006(5)	0.008(6)	0.01(3)
GOOF on F^2	0.816	0.907	1.026	1.035

Table S2. Atomic Coordinates (×10^4) and Equivalent Isotropic Displacement Parameters ($Å^2$×10^3).

Atom	Wyckoff	Site	*x*	*y*	*z*	U(eq)
			**SBMSP**			
Br	2a	2	5000	7046.6(7)	0	18.4(2)
Mg1	2b	2	5000	5601(2)	5000	16.6(7)
Mg2	2b	2	0	7056.1(19)	5000	8.7(6)
P1	4c	1	3574.9(17)	6474.7(11)	2786.4(19)	11.5(4)
P2	4c	1	3712.8(15)	7803.1(11)	5611.3(18)	8.6(4)
P3	4c	1	1339.8(18)	6156.1(13)	4470.2(19)	15.2(4)
P4	4c	1	1085.5(15)	7947.8(11)	1766.6(17)	9.3(4)
P5	4c	1	1262.7(16)	6139.0(12)	-681.2(18)	9.7(4)
P6	4c	1	3790.8(17)	4649.8(12)	676.6(18)	11.7(4)
P7	4c	1	6244.9(16)	4570.7(12)	4227.5(18)	13.1(4)
P8	4c	1	6453.5(18)	2830.2(11)	7007(2)	14.1(4)
P9	4c	1	8700.6(16)	4439.1(11)	8130.5(18)	10.2(4)
P10	4c	1	11162.6(17)	4430.7(12)	7002.8(19)	12.4(4)
Si1	4c	1	2324.2(16)	7092.7(12)	3613.8(18)	9.0(4)
Si2	2b	2	0	7056.1(19)	5000	8.7(6)
Si3	2a	2	0	6954.9(17)	0	8.7(5)
Si4	4c	1	2510.7(17)	5374.4(11)	1341.9(19)	8.6(4)
Si5	4c	1	4842.3(17)	3747.3(12)	2509.1(19)	9.3(4)
Si6	4c	1	7432.6(17)	3734.6(12)	6068.8(19)	8.8(4)
Si7	4c	1	9885.4(18)	5296.6(11)	7431(2)	10.3(4)
Si8	2b	2	10000	3702.4(17)	5000	8.9(5)

Sr1	4c	1	3133.0(7)	8104.2(5)	580.2(7)	21.87(18)
Sr2	4c	1	3284.9(7)	5741.2(6)	-2219.5(8)	26.86(19)
			**SBCSP**			
Br1	2a	2	0	7941.6(6)	0	18.06(17)
Cd1	2b	2	5000	4414.0(4)	5000	13.35(13)
Cd2	2a	2	0	3039.0(12)	0	11.5(4)
Cd3	2b	2	0	2970.7(8)	5000	13.2(3)
P1	4c	1	-1165.4(13)	5557.1(10)	2999.5(15)	11.1(3)
P2	4c	1	1353.1(15)	3845.1(12)	4470.9(16)	16.2(3)
P3	4c	1	1086.8(12)	2054.2(9)	1760.0(14)	8.4(3)
P4	4c	1	1300.4(12)	5552.7(10)	1870.8(14)	9.7(3)
P5	4c	1	1261.5(13)	3859.4(10)	-684.3(14)	8.7(3)
P6	4c	1	3795.8(13)	5338.6(10)	674.2(14)	11.0(3)
P7	4c	1	3575.8(13)	3514.3(10)	2777.5(15)	9.3(3)
P8	4c	1	6273.8(12)	5437.4(10)	4207.3(14)	11.3(3)
P9	4c	1	8717.5(12)	7194.0(9)	5615.4(14)	7.2(3)
P10	4c	1	6455.1(14)	7158.1(10)	7014.8(17)	13.6(3)
Si1	4c	1	116.5(13)	4701.3(11)	2568.5(15)	9.1(3)
Si2	2a	2	0	3039.0(12)	0	11.5(4)
Si3	2b	2	0	2970.7(8)	5000	13.2(3)
Si4	4c	1	2324.8(12)	2903.9(11)	3614.4(14)	7.6(3)
Si5	4c	1	2518.4(13)	4621.3(10)	1344.8(15)	6.9(3)
Si6	4c	1	4841.0(13)	6249.3(11)	2496.0(15)	8.3(3)
Si7	4c	1	7438.6(13)	6266.6(10)	6072.7(15)	7.3(3)
Si8	2b	2	0	6294.7(14)	5000	7.4(4)

Sr1	4c	1	-1859.9(5)	6879.4(4)	583.4(6)	20.58(14)
Sr2	4c	1	6715.3(6)	4234.3(5)	2231.5(6)	25.94(15)
			**BBGSP**			
Ba1	4c	1	6667.4(7)	4738.8(3)	14501.6(8)	14.5(2)
Ba2	4c	1	-1687.4(7)	2921.7(3)	7736.1(8)	16.3(2)
Ba3	4c	1	6047.9(8)	6353.6(4)	8000.8(13)	36.1(3)
Br1	2b	2	5000	5624.6(8)	15000	29.8(5)
Br2	2a	2	0	2083.3(9)	10000	33.7(6)
Ga1	2a	2	5000	4425.0(9)	10000	17.6(5)
Ga2	4c	1	7(3)	6354.6(12)	2512(3)	9.6(5)
Ga3	2a	2	0	7363.3(13)	0	9.7(7)
Ga4	2a	2	0	5334.3(12)	10000	8.1(6)
P1	4c	1	3686(3)	7806.6(11)	3117(3)	8.1(5)
P2	4c	1	1131(3)	7924.3(13)	4362(3)	7.8(5)
P3	4c	1	1291(3)	6836.1(12)	1853(3)	10.5(6)
P4	4c	1	3556(3)	6907.7(14)	5626(4)	13.1(6)
P5	4c	1	1287(3)	6888.6(12)	6966(3)	10.0(6)
P6	4c	1	3584(3)	5806.9(14)	7863(4)	14.8(6)
P7	4c	1	1285(3)	5808.0(13)	4290(3)	10.2(6)
P8	4c	1	1215(3)	4799.3(12)	6792(3)	9.1(6)
P9	4c	1	1279(3)	5888.9(12)	9439(3)	11.0(6)
P10	4c	1	-1257(3)	3780.3(12)	5748(3)	10.4(6)
P11	4c	1	1301(3)	4825.9(12)	11932(3)	8.9(6)
P12	4c	1	3646(3)	4961.6(12)	10611(3)	9.3(6)
P13	4c	1	3762(3)	3922.8(12)	13103(3)	9.5(6)

P14	4c	1	6248(3)	3910.8(12)	12013(3)	8.6(5)
P15	4c	1	6388(3)	2838.8(12)	9437(3)	9.3(6)
P16	4c	1	8732(3)	3786.5(11)	10622(3)	8.1(6)
Si1	2b	2	0	5356.1(17)	5000	6.9(8)
Si2	4c	1	7(3)	6354.6(12)	2512(3)	9.6(5)
Si3	2a	2	0	7363.3(13)	0	9.7(7)
Si4	2a	2	0	5334.3(12)	10000	8.1(6)
Si5	4c	1	2322(3)	7377.6(12)	3761(3)	8.4(6)
Si6	2b	2	0	7346.7(17)	5000	6.5(8)
Si7	4c	1	2344(3)	6351.6(13)	6143(3)	8.9(6)
Si8	4c	1	2338(3)	5366.9(13)	8556(3)	8.9(6)
Si9	4c	1	2507(3)	4347.9(13)	11132(3)	7.5(6)
Si10	4c	1	4859(3)	3397.1(12)	12355(3)	7.5(6)
Si11	4c	1	7427(3)	3373.4(12)	11375(3)	6.8(6)
Si12	2b	2	0	3359.3(18)	5000	9.0(9)
Si13	4c	1	-7(3)	4295.2(12)	7499(3)	7.3(6)
			**BBISP**			
Ba1	4c	1	3958.3(12)	6345.7(6)	7043(3)	93.7(9)
Ba2	4c	1	-1705.0(9)	2929.1(4)	2736.0(10)	30.9(6)
Ba3	4c	1	3324.8(9)	4709.0(4)	500.9(10)	30.2(6)
Br1	2a	2	5000	5589.4(12)	0	68.8(12)
Br2	2b	2	0	2092.1(12)	5000	71.3(13)
In1	2b	2	5000	4445.1(6)	5000	28.2(6)
In2	2b	2	10000	5357.7(7)	5000	16.3(6)
In3	2b	2	10000	7315.6(10)	5000	23.1(8)

P1	4c	1	-1239(4)	3759.9(16)	738(4)	24.7(10)
P2	4c	1	1346(4)	2784.5(17)	1907(4)	27.7(10)
P3	4c	1	1280(4)	3777.5(16)	4353(4)	23.7(9)
P4	4c	1	3605(4)	2831.4(15)	5578(4)	26.4(10)
P5	4c	1	3697(4)	3870.5(16)	2917(4)	23.6(9)
P6	4c	1	6197(4)	3916.0(15)	1921(4)	22.0(9)
P7	4c	1	6466(4)	4989.7(18)	4363(4)	27.0(10)
P8	4c	1	8660(4)	4789.1(15)	2983(4)	25.1(9)
P9	4c	1	8667(4)	5940.4(18)	5632(5)	32.2(11)
P10	4c	1	8776(4)	4797.0(15)	8187(4)	23.8(9)
P11	4c	1	6424(4)	5782.2(19)	7189(5)	33.9(11)
P12	4c	1	8720(4)	6884.1(17)	8068(4)	26.2(10)
P13	4c	1	11307(5)	6812(2)	6942(5)	34.8(12)
P14	4c	1	8738(4)	5809.1(18)	10708(5)	27.1(10)
P15	4c	1	6453(4)	6903.1(19)	9328(5)	32.9(11)
P16	4c	1	8880(3)	7907.5(16)	10651(4)	22.5(9)
Si1	4c	1	7509(4)	4340.3(16)	3869(5)	23.8(10)
Si2	2b	2	10000	5357.7(7)	5000	16.3(6)
Si3	2b	2	10000	7315.6(10)	5000	23.1(8)
Si4	4c	1	10006(4)	6358.4(19)	7572(5)	26.1(10)
Si5	4c	1	7675(4)	5380.7(17)	6460(5)	26.3(10)
Si6	2a	2	10000	5347(2)	10000	15.4(11)
Si7	2a	2	10000	7336(2)	10000	18.3(12)
Si8	4c	1	7648(4)	6344.5(17)	8842(5)	25.3(10)
Si9	4c	1	7683(4)	7361.6(16)	11215(4)	24.1(10)

Si10	4c	1	5122(4)	3379.4(16)	2647(4)	20.6(9)
Si11	4c	1	2556(4)	3349.5(16)	3617(4)	22.2(9)
Si12	2a	2	0	3322(2)	0	17.9(11)
Si13	4c	1	-12(4)	4275.4(16)	2466(4)	21.8(10)

Table S3. Selected bond lengths (Å).

Bond	Length/Å	Bond	Length/Å
**SBMSP**			
Sr1^{1}-Br	3.0063(8)	Si1-P1	2.222(2)
Sr1-Br	3.0063(8)	Si1-P2	2.266(2)
Sr2^{1}-Br	3.0350(9)	Si1-P3	2.253(2)
Sr2-Br	3.0350(9)	Si1-P4	2.238(2)
Mg1-P1	2.536(3)	Si2-P3	2.339(3)
Mg1^{9}-P1	2.536(3)	Si2^{10}-P8	2.346(2)
Mg1-P7	2.511(3)	Si3-P4	2.276(2)
Mg1^{9}-P7	2.511(3)	Si3-P5	2.282(2)
Mg2-P3	2.339(3)	Si4-P1	2.224(2)
Sr1-P1	3.2388(19)	Si4-P5	2.274(2)
Sr1-P4	3.1793(16)	Si4-P6	2.217(2)
Sr1-P5	3.604(2)	Si4^{9}-P9	2.253(2)
Sr1-P6^{3}	3.132(2)	Si5-P6	2.219(2)
Sr1-P4	3.1793(16)	Si5-P7	2.210(3)
Sr1-P8^{2}	3.353(2)	Si5-P8^{9}	2.289(2)
Sr1-P9^{2}	3.5228(18)	Si5^{7}-P4	2.289(2)

Sr1-P10[4]	3.018(2)	Si6[7]-P2	2.274(2)
Sr2-P3[6]	3.2313(19)	Si6-P7	2.201(2)
Sr2-P5	3.4669(19)	Si6-P8	2.272(2)
Sr2-P6[1]	3.540(2)	Si6-P9	2.261(2)
Sr2-P7[1]	2.9585(19)	Si7-P9	2.256(2)
Sr2-P8[2]	3.728(2)	Si7-P10	2.196(3)
Sr2-P10[5]	3.033(2)	Si7[5]-P5	2.302(2)
Si8-P10	2.209(2)	Si7[9]-P3	2.269(3)
Si8-P10[8]	2.209(2)	Si8[7]-P2	2.335(2)
Cd14-As6	2.540(2)		

SBCSP

Sr1[1]-Br1	3.0080(8)	Si1-P1	2.195(2)
Sr1[2]-Br1	3.0079(8)	Si1-P2	2.274(2)
Sr2[3]-Br1	3.0326(9)	Si1-P4	2.252(2)
Sr2[4]-Br1	3.0326(9)	Si1[2]-P5	2.302(2)
Cd1-P7	2.5651(15)	Si2-P3	2.2683(18)
Cd1-P7[1]	2.5651(15)	Si2-P3[2]	2.2683(18)
Cd1-P8	2.5460(15)	Si2-P5	2.2898(18)
Cd1-P8[1]	2.5460(15)	Si2-P5[2]	2.2898(18)
Cd2-P3	2.2683(18)	Si3-P2	2.3314(19)
Cd2-P5	2.2898(18)	Si4-P2	2.251(2)
Cd3-P2	2.331(2)	Si4-P3	2.240(2)

Cd3-P2[11]	2.3314(19)	Si4-P7	2.2261(19)
Cd3-P10[9]	2.3827(17)	Si4[12]-P9	2.2718(19)
Cd3-P10[10]	2.3827(17)	Si5-P4	2.2576(19)
Sr1-P1	3.0203(16)	Si5-P5	2.281(2)
Sr1-P3[3]	3.1821(14)	Si5-P6	2.2151(19)
Sr1-P4[2]	3.5216(15)	Si5-P7	2.229(2)
Sr1-P5[3]	3.6349(17)	Si6-P6	2.222(2)
Sr1-P6[2]	3.1399(17)	Si6-P8	2.213(2)
Sr1-P7[3]	3.2464(16)	Si6-P10[1]	2.287(2)
Sr1-P10[5]	3.3531(18)	Si6[10]-P3	2.2946(19)
Sr2-P1[7]	3.0473(16)	Si7-P4[1]	2.2643(19)
Sr2-P2[1]	3.2119(17)	Si7-P8	2.206(2)
Sr2-P5[6]	3.4715(16)	Si7-P9	2.2723(19)
Sr2-P6	3.5455(18)	Si7-P10	2.270(2)
Sr2-P6[6]	3.2499(16)	Si8-P1	2.2179(18)
Sr2-P8	2.9525(16)	Si8[7]-P9	2.3362(18)
Sr2-P10[8]	3.7198(18)		

BBGSP

Ba1-Br1	3.1584(16)	Si1-P7	2.258(4)
Ba2-Br2	3.1386(16)	Si1-P7[13]	2.258(4)
Ba3-Br1[7]	3.3321(16)	Si1-P8	2.268(4)
Ba3-Br2[8]	3.3605(17)	Si2-P3	2.263(4)

Ba1-P13[1]	3.400(3)	Si2-P5[13]	2.269(4)
Ba1-P13	3.733(3)	Si21-P7	2.268(4)
Ba1-P10[2]	3.284(3)	Si2-P9[13]	2.257(4)
Ba1-P8[3]	3.315(3)	Si3-P3	2.286(3)
Ba1-P14	3.160(3)	Si4-P11	2.308(3)
Ba1-P11[1]	3.428(3)	Si4-P9	2.307(4)
Ba1-P7[3]	3.471(3)	Si5-P1	2.267(4)
Ba1-P6[3]	3.538(4)	Si5-P2	2.237(4)
Ba2-P13[4]	3.360(3)	Si5-P3	2.265(4)
Ba2-P16[5]	3.529(3)	Si5-P4	2.192(4)
Ba2-P10	3.153(3)	Si6-P2	2.254(4)
Ba2-P5[6]	3.389(3)	Si6-P5	2.257(4)
Ba2-P15[5]	3.428(3)	Si7-P4	2.225(4)
Ba2-P2[6]	3.326(3)	Si7-P5	2.237(4)
Ba2-P4[6]	3.429(4)	Si7-P6	2.220(4)
Ba3-P3[9]	3.331(3)	Si7-P7	2.239(4)
Ba3-P9[3]	3.352(3)	Si8-P12	2.259(4)
Ba3-P4	3.237(3)	Si8-P6	2.195(4)
Ba3-P12[3]	3.722(3)	Si8-P8	2.240(4)
Ba3-P6	3.183(3)	Si8-P9	2.256(4)
Ga1-P14[3]	2.348(3)	Si9-P11	2.274(4)
Ga1-P14	2.348(3)	Si9-P12	2.258(4)

Ga1-P12	2.378(3)	Si9-P13	2.208(4)
Ga1-P12	2.378(3)	Si9^{3}-P16	2.264(4)
Ga2-P3	2.263(4)	Si10-P13	2.209(4)
Ga2-P7	2.268(4)	Si10-P14	2.237(4)
Ga3-P3	2.286(3)	Si10^{11}-P2	2.297(4)
Ga3-P3^{10}	2.286(3)	Si10^{3}-P15	2.283(4)
Ga3-P15^{11}	2.300(3)	Si11-P14	2.233(4)
Ga3-P15^{12}	2.300(3)	Si11-P15	2.276(4)
Ga4-P9	2.307(4)	Si11-P16	2.259(4)
Ga4-P9^{4}	2.307(4)	Si11-P1^{14}	2.273(4)
Ga4-P11	2.308(3)	Si12-P10	2.217(4)
Ga4-P11^{4}	2.308(3)	Si12^{8}-P1	2.348(4)

BBISP

Ba1-P11	3.222(5)	Si1-P6	2.225(6)
Ba1-P15	3.226(5)	Si1-P8	2.261(6)
Ba1-P1^{15}	3.355(3)	Si1-P7	2.242(6)
Ba1^{13}-P13	3.347(5)	Si1^{2}-P3	2.279(6)
Ba1^{14}-Br2	3.441(3)	Si2-P8	2.468(4)
Ba1^{2}-P7	3.644(5)	Si2-P9	2.454(5)
Ba1^{2}-P9	3.335(5)	Si3-P13	2.321(6)
Ba1^{6}-Br2	3.441(3)	Si4-P12	2.250(6)
Ba2-P6^{5}	3.365(4)	Si4-P14^{10}	2.243(6)

Ba2-P3^{4}	3.546(4)	Si4-P13	2.244(6)
Ba2-P16^{6}	3.355(4)	Si4-P9	2.223(6)
Ba2-P1	3.153(4)	Si5-P10	2.248(6)
Ba2-P12^{6}	3.414(4)	Si5-P7	2.253(6)
Ba2-P4^{4}	3.429(5)	Si5-P9	2.237(6)
Ba2-P15^{6}	3.441(5)	Si5-P11	2.194(6)
Ba2-Br2	3.155(2)	Si6-P10^{10}	2.282(5)
Ba3-P6	3.689(4)	Si6-P10	2.282(5)
Ba3-P6^{1}	3.439(4)	Si6-P14	2.266(5)
Ba3-P10^{2}	3.349(4)	Si6-P14^{10}	2.266(5)
Ba3-P5	3.151(4)	Si7-P16^{10}	2.257(5)
Ba3-P1^{3}	3.285(4)	Si7-P16	2.257(5)
Ba3-P8^{1}	3.371(4)	Si7-P12	2.237(5)
Ba3-P14^{2}	3.551(5)	Si7-P12^{1}	2.237(5)
Ba3-P11^{2}	3.527(5)	Si8-P12	2.240(6)
Ba3Br1	3.174(2)	Si8-P14	2.253(6)
In1-P5	2.504(4)	Si8-P11	2.216(6)
In1-P5^{2}	2.504(4)	Si8-P15	2.211(6)
In1-P7	2.528(4)	Si9-P16	2.235(6)
In1-P7^{2}	2.528(4)	Si9-P13^{10}	2.241(6)
In2-P8	2.468(4)	Si9-P15	2.200(6)
In2-P8^{7}	2.468(4)	Si9^{12}-P2	2.281(6)

In2-P9	2.454(5)	Si10-P6	2.209(5)
In2-P9^{7}	2.454(5)	Si10-P5	2.219(6)
In3-P4^{8}	2.387(4)	Si10^{11}-P16	2.291(5)
In3-P4^{9}	2.387(4)	Si10^{2}-P4	2.277(6)
In3-P13	2.321(6)	Si11-P3	2.254(6)
In3-P13^{7}	2.321(6)	Si11-P5	2.228(6)
Si12-P2	2.362(5)	Si11-P4	2.280(6)
Si13-P10^{2}	2.287(6)	Si11-P2	2.245(6)
Si13-P3	2.275(5)	Si12-P1^{3}	2.231(5)
Si13-P1	2.185(6)	Si12-P1	2.231(5)
Si13-P8^{5}	2.285(6)	Si12-P2^{3}	2.362(5)

Table S4. Anisotropic Displacement Parameters (Å^2×10^3).

Atom	U_{11}	U_{22}	U_{33}	U_{23}	U_{13}	U_{12}
			**SBMSP**			
Br	15.3(5)	16.7(5)	23.5(5)	0	8.7(4)	0
Mg1	13.4(18)	18.6(19)	14.4(17)	0	3.1(14)	0
Mg2	7.8(13)	9.9(14)	8.5(13)	0	3.7(11)	0
P1	11.6(9)	9.5(9)	11.4(9)	-2.6(7)	3.2(7)	-0.1(7)
P10	10.0(9)	13.3(9)	12.8(9)	-2.3(7)	3.9(7)	0.2(7)
P2	7.3(9)	9.7(9)	9.0(8)	-0.4(7)	3.7(7)	0.2(7)
P3	15.6(10)	14.6(9)	11.7(9)	0.8(8)	2.5(8)	-7.6(8)
P4	8.1(8)	10.3(9)	8.9(8)	0.3(7)	3.3(7)	-0.1(7)
P5	9.6(9)	7.5(8)	8.5(8)	0.1(7)	0.7(7)	1.4(7)
P6	14.4(9)	11.6(9)	10.8(8)	2.8(7)	7.2(7)	5.7(7)
P7	10.5(9)	14.3(10)	11.9(8)	0.2(7)	2.5(7)	1.4(7)
P8	17.2(10)	10.4(9)	21.2(10)	-1.4(8)	14.5(8)	-1.4(7)
P9	9.7(9)	10.6(9)	8.7(8)	-2.1(7)	2.5(7)	0.7(7)
Si1	7.8(9)	10.0(9)	8.5(8)	0.1(8)	2.9(7)	-0.9(8)
Si2	7.8(13)	9.9(14)	8.5(13)	0	3.7(11)	0
Si3	10.4(13)	6.9(13)	8.1(12)	0	3.4(10)	0
Si4	9.4(10)	8.0(9)	7.8(9)	0.1(7)	3.3(7)	0.4(7)
Si5	8.7(9)	9.9(9)	9.8(9)	0.9(7)	4.6(7)	2.2(7)
Si6	7.4(9)	9.1(9)	9.9(9)	-2.3(7)	3.9(7)	-0.4(7)
Si7	11.6(10)	8.5(9)	8.9(9)	-0.3(7)	2.7(8)	0.7(7)
Si8	8.3(13)	9.7(13)	8.8(12)	0	3.9(11)	0

Sr1	28.6(4)	20.0(4)	21.8(4)	3.6(3)	15.5(3)	8.0(3)
Sr2	28.2(4)	34.7(5)	18.7(4)	-11.2(3)	11.2(3)	-11.3(4)
			**SBCSP**			
Br1	15.5(4)	16.5(4)	22.5(4)	0	8.7(3)	0
Cd1	11.8(3)	13.0(3)	12.3(3)	0	2.6(2)	0
Cd2	11.2(8)	11.7(9)	10.9(8)	0	4.1(6)	0
Cd3	12.6(5)	14.3(6)	13.7(5)	0	6.7(4)	0
P1	9.2(6)	11.1(7)	11.5(6)	-3.3(6)	3.3(5)	0.9(6)
P2	16.8(7)	16.4(8)	9.5(7)	-3.5(6)	0.4(6)	9.6(6)
P3	7.6(6)	8.5(7)	9.1(6)	-1.5(6)	3.7(5)	-1.5(5)
P4	9.7(6)	9.5(7)	8.4(6)	-2.4(6)	2.5(5)	0.4(5)
P5	9.3(6)	8.1(7)	7.2(6)	-0.1(5)	2.2(5)	-0.8(5)
P6	13.1(6)	11.6(7)	9.9(6)	-3.0(6)	6.5(5)	-5.6(6)
P7	9.7(6)	7.8(7)	8.7(6)	0.4(5)	2.4(5)	-0.6(5)
P8	9.1(6)	12.5(7)	10.7(6)	-0.8(6)	2.9(5)	-0.5(6)
P9	6.2(6)	7.6(7)	7.8(6)	0.3(5)	3.2(5)	-0.3(5)
P10	16.8(7)	8.7(7)	22.2(7)	0.1(6)	15.0(6)	1.1(6)
Si1	9.6(7)	8.0(7)	8.0(7)	-1.1(6)	2.3(5)	1.7(6)
Si2	11.2(8)	11.7(9)	10.9(8)	0	4.1(6)	0
Si3	12.6(5)	14.3(6)	13.7(5)	0	6.7(4)	0
Si4	6.7(6)	8.1(7)	7.0(6)	-0.3(6)	2.2(5)	0.7(6)
Si5	7.7(6)	6.6(7)	6.8(6)	0.2(5)	3.4(5)	-0.5(5)
Si6	8.0(6)	8.3(7)	8.9(6)	-1.3(6)	4.1(5)	-2.5(6)
Si7	6.7(6)	7.0(7)	8.5(6)	1.0(6)	3.7(5)	0.7(6)
Si8	8.2(9)	7.3(10)	7.5(9)	0	4.3(7)	0

Sr1	28.4(3)	18.3(3)	19.9(3)	-2.8(2)	15.0(2)	-7.4(3)
Sr2	29.4(3)	31.6(4)	18.8(3)	-11.7(3)	12.3(2)	-11.2(3)
			**BBGSP**			
Ba1	16.5(4)	14.7(4)	14.6(4)	-1.5(2)	8.9(3)	0.8(3)
Ba2	18.8(4)	14.7(4)	13.8(4)	0.2(3)	5.6(3)	0.3(3)
Ba3	11.0(4)	28.3(5)	58.3(7)	5.7(4)	4.7(4)	-0.6(3)
Br1	42.3(13)	14.4(9)	51.7(15)	0	38.2(12)	0
Br2	47.3(14)	18.6(10)	21.2(11)	0	1.2(10)	0
Ga1	16.6(11)	18.2(12)	18.4(12)	0	7.8(9)	0
Ga2	12.8(12)	8.3(12)	8.1(13)	-1.3(9)	4.8(10)	0.0(10)
Ga3	11.0(15)	10.9(15)	5.8(15)	0	2.4(12)	0
Ga4	10.3(14)	8.6(14)	6.3(15)	0	4.3(12)	0
P1	11.2(13)	6.6(13)	8.6(14)	-0.9(10)	6.1(11)	-1.5(10)
P2	9.0(12)	8.2(13)	5.6(13)	-0.3(10)	2.4(10)	-0.5(10)
P3	12.4(14)	9.0(14)	8.6(15)	-1.4(10)	3.2(12)	-0.5(11)
P4	8.6(13)	12.9(14)	15.4(15)	8.2(11)	2.7(11)	1.2(11)
P5	11.8(14)	8.6(13)	10.2(15)	0.4(10)	5.2(12)	2.2(11)
P6	9.0(13)	14.2(15)	19.1(16)	9.3(12)	3.9(11)	0.0(11)
P7	13.1(14)	7.6(13)	10.2(14)	0.5(10)	5.3(11)	0.2(11)
P8	9.6(13)	9.5(14)	7.8(14)	1.2(10)	3.4(11)	-1.0(10)
P9	11.7(15)	11.8(15)	8.1(15)	-0.3(10)	2.8(12)	3.8(11)
P10	9.1(14)	11.0(14)	10.0(14)	-2.3(10)	3.0(11)	-0.5(10)
P11	9.2(13)	9.8(14)	8.6(13)	1.5(10)	4.5(11)	2.5(10)
P12	9.8(13)	8.2(14)	8.1(15)	1.4(10)	2.2(11)	-0.8(10)
P13	11.7(14)	9.5(14)	7.3(14)	0.4(10)	4.0(11)	3.5(10)

P14	8.2(13)	7.8(13)	9.9(14)	-0.2(10)	4.0(11)	0.9(10)
P15	10.3(12)	8.1(14)	7.5(14)	-0.1(9)	1.8(10)	-2.1(11)
P16	10.6(14)	6.0(14)	8.5(14)	0.4(9)	5.1(11)	0.9(10)
Si1	8(2)	4.2(18)	6(2)	0	1.3(17)	0
Si2	12.8(12)	8.3(12)	8.1(13)	-1.3(9)	4.8(10)	0.0(10)
Si3	11.0(15)	10.9(15)	5.8(15)	0	2.4(12)	0
Si4	10.3(14)	8.6(14)	6.3(15)	0	4.3(12)	0
Si5	10.8(15)	6.4(14)	8.5(15)	0.5(10)	4.5(12)	0.2(10)
Si6	7.7(19)	4.9(19)	7(2)	0	2.8(16)	0
Si7	9.5(13)	7.4(14)	9.3(15)	1.5(10)	3.4(11)	-0.1(11)
Si8	9.3(15)	7.2(14)	8.7(15)	2.0(10)	2.4(12)	0.7(11)
Si9	7.6(13)	8.9(13)	5.6(14)	-0.6(10)	2.6(11)	1.0(10)
Si10	8.9(14)	9.6(13)	5.2(14)	1.6(10)	4.1(12)	1.3(11)
Si11	7.6(13)	8.2(13)	4.3(13)	1.7(10)	2.3(11)	0.9(10)
Si12	8.2(19)	12(2)	6(2)	0	1.9(16)	0
Si13	6.6(13)	7.7(13)	8.1(15)	-0.4(10)	3.6(11)	-0.9(11)
			**BBISP**			
Ba1	28.6(8)	43.2(9)	171(2)	-18.0(10)	7.0(9)	0.8(6)
Ba2	33.2(7)	30.0(7)	27.8(7)	0.3(4)	11.5(4)	-0.5(4)
Ba3	31.3(7)	31.4(7)	29.0(7)	2.2(4)	14.0(4)	-0.2(4)
Br1	90(3)	32.8(14)	129(3)	0	89(3)	0
Br2	89(3)	37.7(15)	43.5(17)	0	-12.8(17)	0
In1	27.1(9)	27.9(9)	30.0(9)	0	12.6(7)	0
In2	17.2(10)	15.4(9)	15.9(10)	0	7.0(8)	0
In3	23.5(14)	24.4(14)	20.9(13)	0	9.1(11)	0

P1	27(2)	23.6(19)	22.8(19)	-1.7(15)	10.0(17)	-1.1(16)
P2	29(2)	35(2)	23(2)	5.8(16)	14.6(16)	12.5(17)
P3	23(2)	24.1(19)	24(2)	-1.0(15)	10.7(16)	-1.0(15)
P4	29(2)	21(2)	25.6(19)	2.0(14)	9.0(16)	2.1(15)
P5	27(2)	22.4(18)	24.1(19)	0.6(15)	13.3(16)	1.0(16)
P6	23(2)	20.8(18)	22.2(19)	-0.3(14)	9.3(16)	-5.2(14)
P7	24(2)	37(2)	18(2)	-3.3(15)	7.6(16)	-5.3(16)
P8	29(2)	23(2)	25.8(19)	0.1(15)	14.3(16)	-2.6(16)
P9	33(2)	38(2)	23(2)	-3.1(17)	9.2(18)	-11.0(18)
P10	26(2)	22(2)	24.7(19)	-2.2(15)	12.6(15)	-1.7(15)
P11	27(2)	31(2)	40(2)	-8.9(19)	10.4(18)	0.5(18)
P12	27(2)	24.3(19)	25(2)	2.8(16)	8.1(17)	-0.4(16)
P13	38(3)	41(3)	32(2)	-10.1(19)	21(2)	-10(2)
P14	27(2)	24.2(18)	32(2)	3.2(16)	14.5(18)	0.4(17)
P15	27(2)	29(2)	42(3)	-9.4(18)	14.4(19)	-3.0(17)
P16	22.7(18)	21.5(17)	21.6(17)	-1.1(15)	8.0(14)	-1.5(16)
Si1	23(2)	23.0(19)	25(2)	-0.1(15)	9.1(16)	-0.5(15)
Si2	17.2(10)	15.4(9)	15.9(10)	0	7.0(8)	0
Si3	23.5(14)	24.4(14)	20.9(13)	0	9.1(11)	0
Si4	31(2)	22.3(18)	26(2)	-0.6(16)	13.1(17)	-6.2(17)
Si5	28(2)	24.5(19)	28(2)	-0.3(16)	13.0(18)	-0.4(17)
Si6	16(3)	13(2)	15(2)	0	4(2)	0
Si7	19(3)	18(2)	19(3)	0	10(2)	0
Si8	27(2)	19.7(18)	29(2)	-1.0(16)	12.5(17)	-3.0(17)
Si9	25(2)	21.5(18)	26(2)	-0.1(16)	11.1(17)	0.4(16)

Si10	23(2)	19.7(18)	19.9(19)	-1.7(15)	9.9(16)	-3.5(15)
Si11	21(2)	21.9(18)	23(2)	-0.6(15)	8.8(16)	2.1(15)
Si12	11(2)	26(3)	13(2)	0	1.8(19)	0
Si13	22(2)	19.3(18)	23(2)	1.6(15)	8.9(17)	0.8(15)

Table S5. The dipole moments of groups within one unit cell.

Groups	μ_x	μ_y	μ_z	μ_{total}
		**SBMSP**		
$[SiP_4]1\times4$	0	0.0453	0	0.181317595
$[Si/MgP_4]2\times2$	0	-3.911	0	-7.821
$[SiP_4]3\times2$	0	5.852	0	11.705
$[SiP_4]4\times4$	0	3.584	0	14.337
$[SiP_4]5\times4$	0	1.309	0	5.237
$[SiP_4]6\times4$	0	-4.494	0	-17.975
$[SiP_4]7\times4$	0	-1.295	0	-5.181
$[SiP_4]8\times2$	0	-4.006	0	-8.013
$[MgP_4]1\times2$	0	-3.455	0	-6.910
Total	0	-14.439	0	-14.439
		**SBCSP**		
$[SiP_4]1\times4$	0	-3.780670529	0	-15.12268212
$[Si/CdP_4]2\times2$	0	-5.550	0	-11.099
$[Si/CdP_4]3\times2$	0	2.374	0	4.748
$[SiP_4]4\times4$	0	0.044	0	0.177
$[SiP_4]5\times4$	0	-3.781	0	-15.123
$[SiP_4]6\times4$	0	-1.277	0	-5.107
$[SiP_4]7\times4$	0	4.260	0	17.042

$[SiP_4]8\times2$	0	0	0	0.000
$[CdP_4]1\times2$	0	2.033	0	4.066
Total	0	-20.418	0	-20.418
**BBGSP**				
$[SiP_4]1\times4$	0	-5.697589382	0	-22.79035753
$[Ga/SiP_4]2\times2$	0	0.048	0	0.096
$[Ga/SiP_4]3\times2$	0	-2.691	0	-5.383
$[Ga/SiP_4]4\times4$	0	2.115	0	8.462
$[SiP_4]5\times4$	0	-1.369	0	-5.474
$[SiP_4]6\times2$	0	6.869	0	13.738
$[SiP_4]7\times2$	0	0.138	0	0.276
$[SiP_4]8\times4$	0	-0.021	0	-0.085
$[SiP_4]9\times4$	0	2.635	0	10.540
$[SiP_4]10\times4$	0	1.229	0	4.917
$[SiP_4]11\times4$	0	-3.831	0	-15.322
$[SiP_4]12\times2$	0	-5.361	0	-10.723
$[SiP_4]13\times4$	0	-0.383	0	-1.533
$[GaP_4]1\times2$	0	0.286	0	0.572
Total	0	-22.710	0	-22.710
**BBISP**				
$[SiP_4]1\times4$	0	3.251340425	0	13.0053617
$[In/SiP_4]2\times2$	0	0.763	0	1.526
$[In/SiP_4]3\times2$	0	-0.621	0	-1.242
$[SiP_4]4\times4$	0	0.119	0	0.476
$[SiP_4]5\times4$	0	0.173	0	0.694

$[SiP_4]6\times2$	0	-4.557	0	-9.113
$[SiP_4]7\times2$	0	6.716	0	13.432
$[SiP_4]8\times4$	0	0.168	0	0.673
$[SiP_4]9\times4$	0	-1.701	0	-6.803
$[SiP_4]10\times4$	0	1.263	0	5.051
$[SiP_4]11\times4$	0	-3.595	0	-14.380
$[SiP_4]12\times2$	0	-3.525	0	-7.050
$[SiP_4]13\times4$	0	-0.245	0	-0.980
$[InP_4]1\times2$	0	-1.438	0	-2.877
Total	0	-7.587	0	-7.587

Table S6. Calculated SHG coefficients.

d_{ij}	SBMSP	SBCSP	BBGSP	BBISP
d_{21}	49	38	37	43
d_{25}	57.5	58	63	80
d_{23}	64.8	76	71	90
d_{22}	4.6	6.8	2.3	2.5

Table S7. Powder Laser-Induced Damage Threshold (LIDT) Measurements.

	Energy (mJ)	Spot area (cm^2)	Pulse width (ns)	Repetition frequency (Hz)	LIDT (MW/cm^2)
SBMSP	28.4	0.1	10	1	28.4
SBCSP	27.5	0.1	10	1	27.5
BBGSP	27.3	0.1	10	1	27.3
BBISP	26.7	0.1	10	1	26.7
$AgGaS_2$	3.1	0.1	10	1	3.1

Table S8. Band gaps of representative A-M-Pn phosphides.

Phosphides	Band gap (eV)	References	Phosphides	Band gap (eV)	References
Li_2SiP_2	2.32[a]		$MgSiP_2$	2.34	19
Li_2GeP_2	2.2[a]		α-Ca_2CdP_2	1.98	43
$Ca_2Li_4SiP_4$	2.15[a]		$SrSi_7P_{10}$	1.51	31
Na_2GeP_2	1.98[a]		Sr_2SiP_4	1.41	44
$NaGe_3P_3$	2.06	34	$SrIn_3Si_4P_9$	1.15	32
KSi_2P_3	1.72[a]		$BaSi_7P_{10}$	1.48	31
K_4ZnP_2	1.58[a]		$Ba_2Si_3P_6$	1.88	33
K_2SiP_2	1.78[a]		$BaCd_2P_2$	1.38[a]	
$RbZn_4P_3$	1.44[a]		Ba_2SiP_4	1.4	44
Rb_3GaP_2	1.65[a]		$BaGe_7P_{12}$	1.6	45
Rb_5GeP_3	1.69[a]		$BaGe_2P_2$	1.36	30
Cs_2SiP_2	1.76[a]		Ba_3GaP_3	1.55[a]	

[a]Calculated by HSE06 in this work

Table S9. Bond populations, Flexible index, SHG and birefringence atomic contributions.

Bonds	Populations	Flexible index	Atoms	SHG contri. (×Si atom)	d_{ij}	Bire. contri. (×Si atom)	Δn
SBMSP					64.8		0.030
Mg-P	0.45	0.262	Mg	0.35		0.23	
Si-P	0.68	0.266	Si	1		1	
			P	2.83		2.36	
SBCSP					76.2		0.036
Cd-P	0.39	0.274	Cd	1.32		1.08	
Si-P	0.68	0.267	Si	1		1	
			P	2.81		2.32	
BBGSP					71.3		0.033
Ga-P	0.53	0.267	Ga	1.13		1.04	
Si-P	0.68	0.266	Si	1		1	
			P	2.78		2.31	
BBISP					89.1		0.051
In-P	0.56	0.303	In	1.47		1.29	
Si-P	0.68	0.268	Si	1		1	
			P	2.74		2.26	

Other Supplementary Materials captions:

Data S1. Crystallographic Information File of $[Sr_4Br]_2[Mg_3Si_{25}P_{40}]$

Data S2. Crystallographic Information File of $[Sr_4Br]_2[Cd_3Si_{25}P_{40}]$

Data S3. Crystallographic Information File of $[Ba_3Br][GaSi_{10}P_{16}]$

Data S4. Crystallographic Information File of $[Ba_3Br][InSi_{10}P_{16}]$
